# Hotspots within a global biodiversity hotspot - areas of endemism are associated with high mountain ranges

**DOI:** 10.1038/s41598-018-28504-9

**Published:** 2018-07-09

**Authors:** Jalil Noroozi, Amir Talebi, Moslem Doostmohammadi, Sabine B. Rumpf, Hans Peter Linder, Gerald M. Schneeweiss

**Affiliations:** 1 Department of Botany and Biodiversity Research, University of Vienna, Vienna, Austria; 2 Department of Plant Science, University of Tehran, Tehran, Iran; 3 Institute of Systematic and Evolutionary Botany, University of Zürich, Zürich, Switzerland

## Abstract

Conservation biology aims at identifying areas of rich biodiversity. Currently recognized global biodiversity hotspots are spatially too coarse for conservation management and identification of hotspots at a finer scale is needed. This might be achieved by identification of areas of endemism. Here, we identify areas of endemism in Iran, a major component of the Irano-Anatolian biodiversity hotspot, and address their ecological correlates. Using the extremely diverse sunflower family (Asteraceae) as our model system, five consensus areas of endemism were identified using the approach of endemicity analysis. Both endemic richness and degree of endemicity were positively related to topographic complexity and elevational range. The proportion of endemic taxa at a certain elevation (percent endemism) was not congruent with the proportion of total surface area at this elevation, but was higher in mountain ranges. While the distribution of endemic richness (i.e., number of endemic taxa) along an elevational gradient was hump-shaped peaking at mid-elevations, the percentage of endemism gradually increased with elevation. Patterns of endemic richness as well as areas of endemism identify mountain ranges as main centres of endemism, which is likely due to high environmental heterogeneity and strong geographic isolation among and within mountain ranges. The herein identified areas can form the basis for defining areas with conservation priority in this global biodiversity hotspot.

## Introduction

A major goal in conservation biology is to determine areas of rich biodiversity^1^. At the global scale, conservation priorities are well established as 34 biodiversity hotspots^2^, i.e. areas featuring exceptional concentrations of endemic species as well as experiencing extreme loss of habitat^3^. These hotspots are, however, at a spatial scale too coarse for conservation management and identification of hotspots at a finer scale, “hotspots-within-hotspots”^1^, is needed to allow comprehensive protection management^1,4,5^. Additionally, biodiversity hotspots are excellent areas to study drivers and processes of diversification. As endemic species are well suited to recognize biodiversity hotspots that also harbour highly threatened species^3,6,7^, identifying areas of endemism (AEs) is an essential part of planning regional conservation management.

AEs are fundamental entities of analyses in biogeography^8^ and are defined as areas of non-random distributional congruence among taxa^9^, whose biogeographical histories may have been affected by common factors, such as geological, ecological or evolutionary processes^8,10^. Whereas biogeographers and evolutionary biologists focus on explaining the causes for the occurrence of AEs^11–13^, ecologists are interested in centres of endemism due to their importance in devising conservation priorities^3,14–17^, which is valuable when financial resources for conservation are limited^3,18,19^. Despite the acknowledged importance of AEs, they remain under-investigated even in currently recognized biodiversity hotspots.

**Figure 1:**
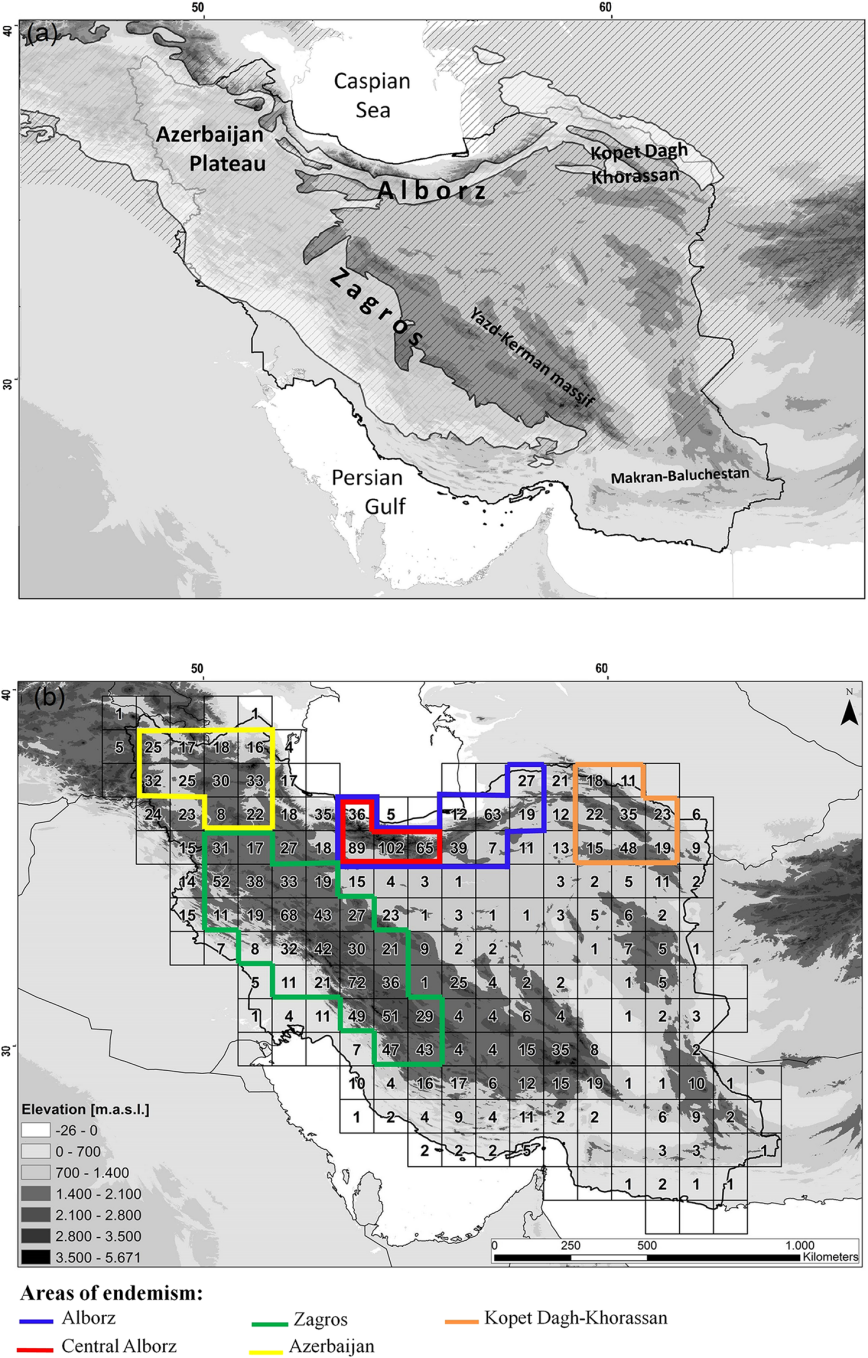
The study area (Iran) and patterns of endemism. (**a**) About half of the study area belongs to the Irano-Anatolian biodiversity hotspot (whitish area). Biogeographically, the study area belongs mainly to the Irano-Turanian region (dashed area), bounded by the Euro-Siberian and the Saharo-Sindian regions in the north and south, respectively. Major mountain ranges are indicated. (**b**) Endemic richness of Asteraceae per grid cell, only taking species within the study area into account, and identified AEs (outlined in colour).

The Irano-Anatolian biodiversity hotspot, which mainly covers high elevations of central and eastern Turkey, Armenia, NE Iraq and Iran, is the only global biodiversity hotspot entirely inside South-West Asia (Fig. 1a). This region has a dry climate with a Mediterranean precipitation regime^20^. It is estimated that more than 40% of the plant species are endemic to this region^2^. Iran covers 54% of the surface area of the Irano-Anatolian hotspot (Fig. 1a), and harbours high plant and animal as well as landscape diversity^21–23^. The Iranian flora comprises more than 8,000 vascular plant taxa, of which about 30% are endemics^24^. Several studies have focused on patterns of endemism, chorology and distribution of vascular plants in Iran^24–36^, providing hypotheses on the locations of putative AEs. For instance, ref.^25^ identified an Alborz distribution pattern (in the Alborz mountains), a Zagros distribution pattern (in the Zagros mountains), and an Armeno-Kurdic distribution pattern (Iranian Azerbaijan and Kurdistan in north-western Iran, north-eastern Iraq, south-eastern Turkey, and Armenia), all of which indicate regions that may, at least partially, constitute AEs. These have, however, never been investigated using a formal quantitative approach.

Here we analyse patterns of endemic richness and identify AEs and their environmental correlates in Iran, using the sunflower family (Asteraceae) as our model group. This family is particularly well suited for this analysis as its members (i) constitute a significant proportion of the Iranian flora in general (16%) and of the endemic flora in particular (23%)^24^, (ii) collectively are distributed over the entire study region and occur in all major habitat types, and (iii) have their taxonomy and distribution well worked out in the Flora Iranica^37^ and the Flora of Iran^38^. We applied endemicity analysis^39^, which has been successfully used in many regions of the world^40–46^ and has been shown to outperform other commonly used methods of defining AEs^47–50^, such as parsimony analysis of endemicity^9^ and biotic element analysis^51^. We addressed the following questions: (1) Where are the main AEs (inferred from patterns of endemic taxon richness and from endemicity analysis) within the study region? Are these exclusively found within the Irano-Anatolian hotspot or also elsewhere? Are these congruent with areas identified previously in phytogeographical studies? (2) What are the major ecological and/or evolutionary factors affecting the distribution of endemics? Specifically, we tested the hypothesis that topographical heterogeneity as a driver of species richness also positively affects endemic richness^52–55^. Additionally, as geographic isolation is an important driver of speciation^56^, we predicted that endemicity increases along an elevational gradient, as geographical isolation increases along this gradient. Finally, species richness decreases with increasing climatic stress^57^ and should therefore result in a hump-shaped pattern of (endemic) species richness along an elevational gradient^58^ due to high environmental stress at xeric lower and cold higher elevations.

## Results

**Figure 2:**
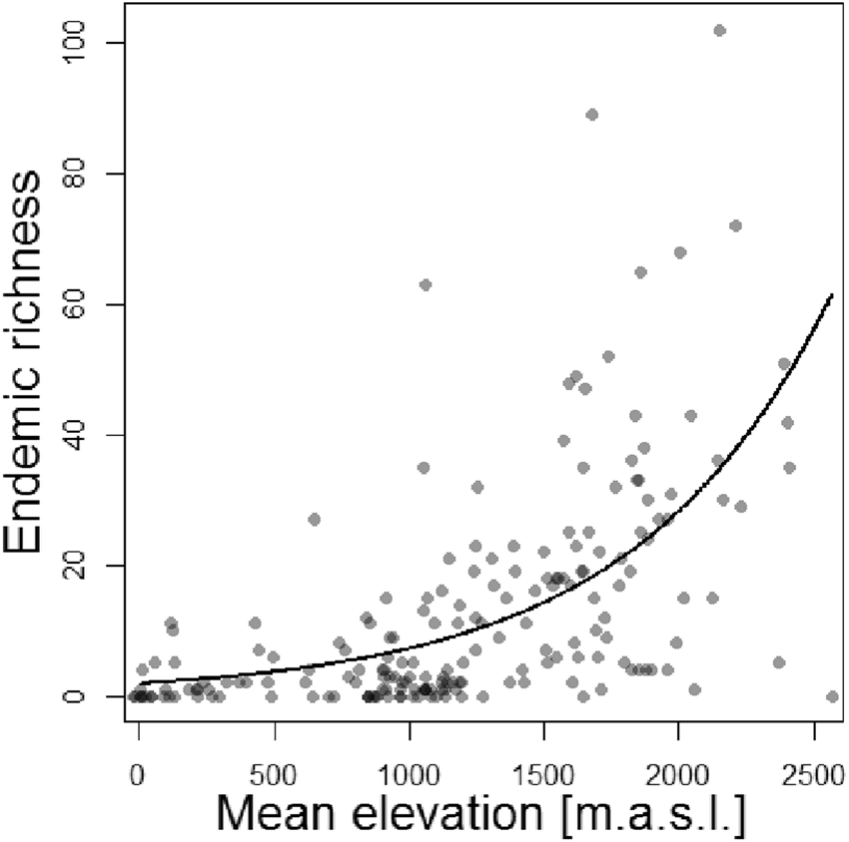
Relation between endemic richness of Iranian Asteraceae and mean elevation (fitted using a generalized linear model with quasi-Poisson distribution and a logarithmic link function).

The 626 endemic and subendemic Asteraceae taxa belonged to 57 genera. Of those, *Cousinia* was the largest genus in the dataset with 229 taxa, followed by *Echinops* (65 taxa), *Centaurea* (64 taxa), *Jurinea* (24 taxa), *Scorzonera* (24 taxa), *Anthemis* (22 taxa) and *Tanacetum* (21 taxa). Most of these species were distributed in the main mountain ranges of Iran. The range sizes of endemic taxa were between one and 51 cells. The endemic richness of cells ranged from zero to 102 taxa per cell (zero to 16.25% of total endemic richness; Fig. 1b). Endemic richness was significantly related to the mean elevation of a grid cell (Table 1). Thus, high richness was observed in high mountains (Fig. 2, Table 1), the highest endemic richness overall corresponding to Central Alborz (Fig. 1b).

**Table 1:** Relations between endemism and environmental variables.

		Regression model^a^	df.^b^	t-value	p-value	Slope ± SE	Intercept ± SE
Endemic richness	Mean elevation [m a.s.l.]	qP	193	9.99	<0.001	1.37^e-3^ ± 0.14^e-3^	0.61 ± 0.23
Endemicity score	Elevational amplitude [m]	qP	185	10.24	<0.001	6.37^e-4^ ± 0.62^e-4^	−5.76^e-2^ ± 0.18^e-2^
Endemicity score	Topographic complexity	qP	185	8.28	<0.001	21.18 ± 2.56	−20.36 ± 2.65
Endemic richness	Elevational amplitude [m]	qP	185	12.14	<0.001	7.52^e-4^ ± 0.62^e-4^	−0.83 ± 0.18
Endemic richness	Topographic complexity	qP	185	9.17	<0.001	24.01 ± 2.62	−22.09 ± 2.71
Percentage of endemism	Elevation [m a.s.l.]	li	41	9.43	<0.001	0.16^e-1^ ± 0.02^e-1^	21.68 ± 4.30

^a^qP, generalized linear model with quasi-Poisson distribution and logarithmic link function; li, linear model.

^b^Degrees of freedom.

The endemicity analysis identified 27 sets (candidate AEs), which were grouped into five consensus areas with maximum scores between 5.72 and 18.20 (Fig. 1b, S1.2, see Appendix S1 in Supporting Information). These five areas were associated with the major mountain ranges (t-test of mean elevation of grid cells belonging to AEs versus mean elevation of grid cells not belonging to AEs: df = 163, t = 7.34, p < 0.001) of the study area. All AEs were associated with the Irano-Anatolian region (Fig. 1).

Alborz: Containing three sets, it comprised 10 grid cells covering central and eastern Alborz (Fig. 1b, S1.2a). The endemicity scores of the area ranged from 6.52 to 11.72. The area was supported by 26 taxa contributing to the score, of which eight (31%) were from *Cousinia*.

Central Alborz: This area was embedded within the previous area, containing a single set only covering the high mountains of Central Alborz (Fig. 1b, S1.2b). Here, the overall highest endemicity score, 17.99, of all recognized areas was found. This area was supported by 24 taxa, which were mostly subalpine and alpine elements, thus restricted to above 2,500 m (Fig. S1.3). Ten of those taxa (41.5%) were from *Cousinia*.

Zagros: Being the biggest of the identified areas, it contained 17 sets covering 23 cells in the Zagros mountains (Fig. 1b, S1.2c). It had scores between 5.73 and 10.59 and was supported by 69 taxa, of which 31 (44.9%) were from *Cousinia*.

Azerbaijan Plateau: Containing four sets, it comprised 10 cells in NW Iran, thus covering the mountain ranges Sabalan, Sahand, Bozqush, Mishodagh and Belqeis (Fig. 1b, S1.2d). It had scores between 7.98 and 8.73 and was supported by 17 taxa, of which seven (41%) were from the genus *Cousinia*.

Kopet Dagh-Khorassan: Containing two sets, it comprised eight cells in the Kopet Dagh and Khorassan mountains in NE Iran (Fig. 1b, S1.2e). The endemicity score ranged from 9.15 to 9.9 and was supported by 19 taxa, of which 10 were from the genus *Cousinia* (53%).

**Figure 3:**
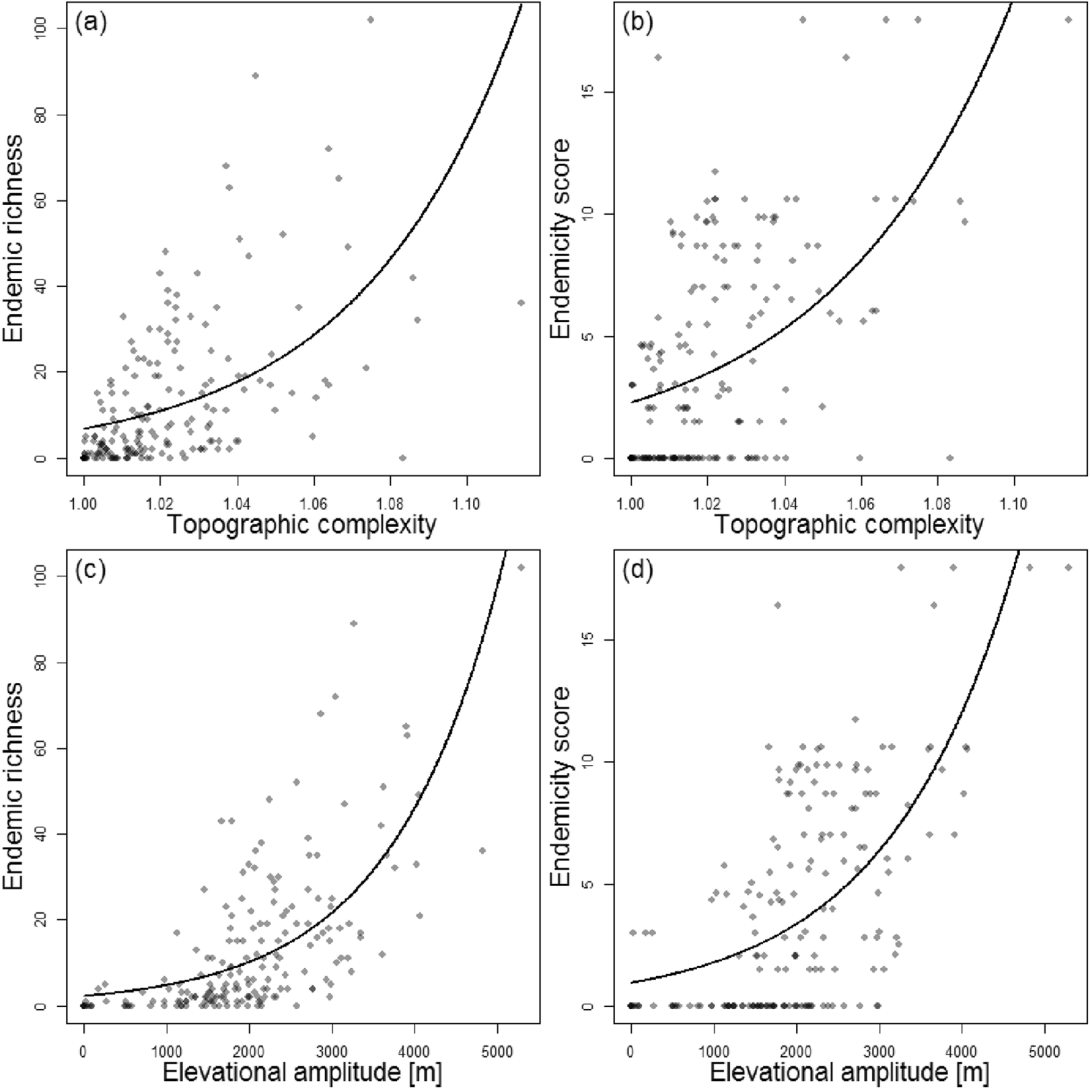
Relations between endemism in Iranian Asteraceae and environment (fitted using generalized linear models with quasi-Poisson distribution and a logarithmic link function). Endemism is measured via (**a**,**c**) endemic species richness or (**b**,**d**) endemicity score, environment is described by (**a**,**b**) topographic complexity and by (**c**,**d**) elevational amplitude.

**Figure 4:**
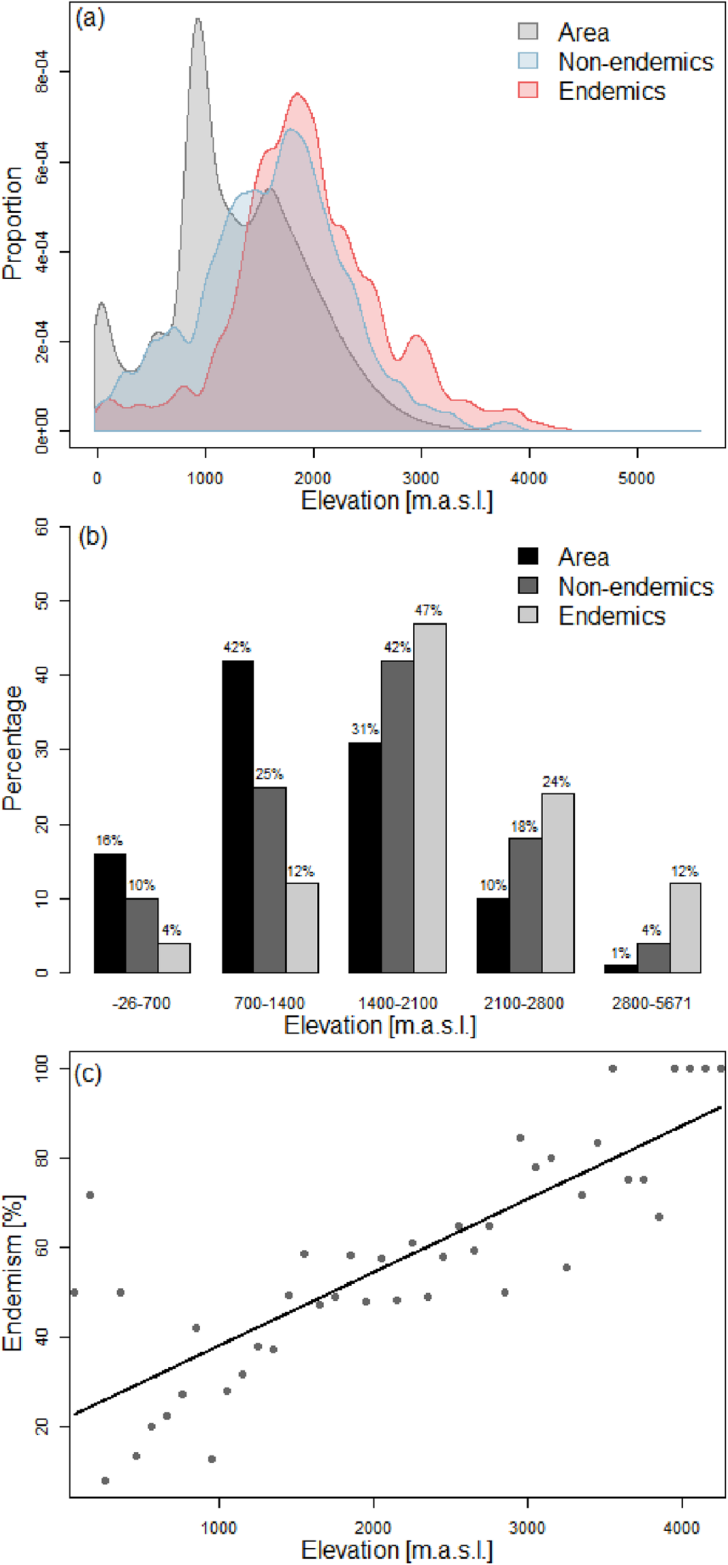
Elevational distributions of endemic compared to non-endemic Asteraceae. (**a**) Proportion of total surface area, percent non-endemism and percent endemism along the elevational gradient. (**b**) Percentage of surface area, non-endemic and endemic richness in different elevational zones. (**c**) Percentage of endemism along the elevational gradient.

Endemic Asteraceae richness and AEs were associated with mountain ranges (Fig. 1), and both endemic richness and degree of endemicity (endemicity score) were positively related to topographic complexity (Fig. 3, Table 1). The proportion of total surface area at a certain elevation declined much more rapidly than the proportions of both non-endemic taxa (percent non-endemism; t-test, df = 637, t = 12.14, p < 0.001) and endemic taxa at this elevation (percent endemism; t-test, df = 623, t = 22.90, p < 0.001; Fig. 4a,b). Moreover, the elevational distribution of the percent endemism was significantly higher than that of the percent non-endemism (t-test, df = 1254, t = 8.34, p < 0.001; Fig. 4a,b). Proportional surface area and endemic richness were not congruent across the elevational gradient (Fig. 4a). While both distributions were hump-shaped, the surface area peaked between 900 and 1,100 m a.s.l., and endemic richness at 1,900 m a.s.l. Specifically, relative to surface area, endemic taxa were underrepresented in lowlands (−26–1,400 m a.s.l.), proportionally represented in mid-elevations (1,400–2,100 m a.s.l.), and overrepresented in high elevations (>2,100 m a.s.l.; Fig. 4a,b). As a corollary, the percentage of endemic taxa increased along the elevational gradient to reach 100% in the subnival zone (Fig. 4c, Table 1). A qualitatively similar distribution was found for non-endemic richness, yet less strongly pronounced than for endemic taxa, i.e. a less severe underrepresentation at lower elevations and a weaker overrepresentation at high elevations (Fig. 4a,b).

## Discussion

Using data from Asteraceae endemic to Iran, we identified five AEs (Fig. 1b). These were exclusively found in the high mountain regions of Iran and were all associated with the Irano-Anatolian hotspot (Fig. 1), thus representing “hotspots-within-hotspots”^1^. The lack of any identified AE outside the Irano-Anatolian hotspot, although it only covers about half of the study area, supports the recognition of this global hotspot. Endemism was correlated with environmental heterogeneity, measured as topographic complexity and elevational amplitude (Fig. 3, Table 1). Whereas the diversity of both endemic and non-endemic Asteraceae peaked at mid elevations, resulting in a hump-shaped distribution of diversity along the elevational gradient (Fig. 4a,b), the percentage of endemic taxa increased continuously with elevation (Fig. 4c).

### AEs in Iran

Of the five identified AEs, four correspond to previously recognized phytogeographic units or distribution patterns. Specifically, the Alborz distribution pattern^26,31,59^ is reflected by the Alborz AE; the Zagros distribution pattern^25,26,29–31,60,61^ and the Kurdo-Zagrosian phytogeographic subprovince^60^ are reflected by the Zagros AE; the Armeno-Kurdic distribution pattern^26,30,60^, although extending beyond the borders of our study region, is reflected in the Azerbaijan Plateau AE; the Kopet Dagh-Khorassan phytogeographic province^35,60^ is reflected by the Kopet Dagh-Khorassan AE. The fifth identified AE, the Central Alborz AE, is geographically embedded within the Alborz AE and as such has not been identified as separate unit before, emphasizing the importance of using methods, such as endemicity analysis, capable of detecting nested AEs. The Central Alborz has many alpine and subnival habitats and almost 70% of the taxa supporting the Central Alborz AE are high elevation species (optimum elevation above 2,500 m; Fig. S1.3).

As the program used for endemicity analysis, NDM/VNDM, does not evaluate and score single cells as putative AEs^48^, putative AEs that are too small relative to grid cell size will remain undetected. This is the case for the high mountains of Yazd-Kerman (35 taxa in one cell of the Hezar-Lalezar Mts. and 27 taxa in the Shirkuh Mts.; Fig. 1b; see also ref.^62^), where most of the endemic taxa are only recorded from one or a few cells. Additional data (including also taxa from other families) as well as alternative approaches (using smaller grid cell size and/or other algorithms, such as sympatry networks)^63^ would help getting a more detailed description of AEs in this region.

### High mountains are centres of endemism

Both patterns of endemic richness as well as AEs inferred via endemicity analysis (Fig. 1b) identify mountain ranges as main centres of endemism in Iran. This is consistent with theory that predicts both increased speciation and reduced extinction rates for mountains^64^. There are many known cases of clades rapidly diversifying in mountains^65^, but this does not prove that endemism in mountains is higher than in the lowlands. Although there is no global compilation to test whether endemism in montane areas is higher than in the surrounding lowlands, there are several case studies demonstrating such a pattern. For example, ref.^66^ showed that the endemism of Mexican monocot geophytes was highest in montane regions, ref.^67^ demonstrated that the New Zealand angiosperm species level endemism is highest in the mountains of South Island. The restriction of narrow-range endemics to mountains appears to be even better developed in the Iberian Peninsula^68^. The Iranian pattern, with the endemic species largely restricted to mountains, is yet another case study consistent with what is probably a global pattern^66–68^.

Topographic heterogeneity is a key environmental predictor of species richness^52–55^. As increased topographic heterogeneity and complexity is expected to result in increased environmental heterogeneity^55,69,70^ the observed pattern is in line with previous hypotheses. High topographic complexity likely causes high habitat diversity and thus a large local niche space^71,72^. This is expected to foster adaptation to different niches (i.e., ecological speciation)^73^ and *in situ* speciation as suggested for Irano-Turanian high mountain ranges^74^ as well as to create local refugia for species during climatic fluctuations reducing extinction risks^75,76^.

Higher diversity in mountain ranges is also expected as a result of allopatric speciation facilitated by strong geographic isolation. Based on dated molecular phylogenies, it has been suggested that the main uplift of the Iranian plateau and the formation of high mountains accelerated during the middle to late Miocene (15–5 Ma) promoted allopatric speciation^77–80^. Many species may have become geographically isolated in high mountains of the Irano-Turanian region during interglacial periods, resulting in disjunct distributions especially at high elevations^25,26,31,33^, further fostering allopatric speciation^81^. Allopatric speciation and ecological speciation are not mutually exclusive hypotheses, and likely both evolutionary processes have contributed to the high biodiversity of the Iranian high mountains.

The highest richness of both endemic as well as non-endemic taxa was found at mid-elevations (hump-shaped distribution). This pattern was first proposed by ref.^82^. Mid-elevation diversity peaks have also been found in a global analysis of geometrid moths^83^ and of ferns^84^. The Iranian Asteraceae pattern suggests that high environmental stress at xeric lower and cold higher elevations causes diversity to peak at mid-elevations^58^. A diversity peak at intermediate elevations has been identified previously for Central Alborz using standardized sample plot data^85^. Although richness distributions for both endemic and non-endemic taxa are hump-shaped, the distribution of endemic taxa is significantly shifted towards higher elevations (Fig. 4a,b). This likely reflects the fact that the percentage of endemism continues to increase with increasing elevation (Fig. 4c). This is in line with observations in other regions^56^ and supports the hypothesis that increasing geographical isolation (not quantified here, but evidently increasing with increasing elevation) at higher elevations positively correlates with the degree of endemicity.

### Conservation implications

Although the high mountain ranges of the study area have already been identified as belonging to the Irano-Anatolian biodiversity hotspot^2^, we could recognize hotspots-within-hotpots using biogeographical analyses of AEs. All five AEs identified in this study have high conservation priority. They are generally rich, also in non-endemics, so that endemic species could serve as flagship species. While resources are probably too limited to protect all parts of the hotspot, focusing on the richest parts of these AEs would help to conserve a high number of threatened endemic species. In spite of low species richness at high elevations compared to mid-elevations, they harbour a high number of endemic and narrowly distributed species in a small area (c. 25% of Iranian endemics are above 2500 m a.s.l.)^24^, where the species are endangered from ongoing climate change^86,87^ and overgrazing effects^88,89^, which warrants a high conservation priority of these habitats.

## Methods

### Study area

Iran is topographically complex (Fig. 1a) due to its location at the interface between the Arabian and Eurasian plates^90,91^. The elevation ranges from 26 m below sea level at the shore of the Caspian Sea to 5,671 m above sea level in Central Alborz. Being part of the Alpine-Himalayan orogenic system^91,92^, the uplift of SW Asian mountain ranges took place between Late Oligocene and Late Miocene^81^. Major mountain ranges in Iran include Zagros in the south-west, Azerbaijan Plateau in the north-west, Alborz in the north, Kopet Dagh-Khorassan in the north-east and east, the Yazd-Kerman massif in the south and the Makran and Baluchestan mountains in the south-east, jointly embracing the central Iranian high plateau (Fig. 1a). Rainfall ranges from less than 25 mm mean annual precipitation in the central deserts up to 2,000 mm in Hyrcanian forests at the northern slopes of Alborz^93^. According to the Global Bioclimatic Classification System^94,95^ there are three macrobioclimates in Iran: Mediterranean (major parts of Iran), tropical (southern Iran) and temperate (northern Iran). These macrobioclimates correlate with the Irano-Turanian, Saharo-Sindian and Euro-Siberian biogeographical regions (Fig. 1a), respectively^93^. Pleistocene climatic fluctuations affected the flora and vegetation of the region^96,97^, causing elevational shifts of vegetation belts or shifts in biomes as a consequence of modified climate zones, e.g., altered boundaries of the inter-tropical convergence zone in the south and south-east or variations in the relative strength of mid-latitude circulation systems^98^. However, knowledge about the evolutionary impact of these climatic oscillations on the flora of this region is still limited.

We restricted the study area to the political border of Iran because floristic records from neighbouring countries are not available in sufficient density. This might introduce edge effects where similar habitats extend beyond the country border, such as lowland habitats in the north-east and east or mountainous habitats in the north-west. However, we argue that the introduced bias will be low, because the majority of Iranian endemic species are restricted to mountain habitats^24,26^, where continuity with areas outside the study region is less extensive than for lowland habitats.

### Distributional data

We selected Asteraceae as our target group because it is the most diverse family in the Iranian flora: it has the largest number of genera, and is exceeded in species richness only by Fabaceae, mainly because of the single genus *Astragalus* with approximately 800 species. About 40% of the Iranian Asteraceae taxa are endemic^24^ and jointly are found in all environments across all elevational zones. The data set comprises 626 of these endemic and subendemic taxa (552 species, 48 subspecies, 26 varieties; Table S1.1). Using information from available floristic literature, we defined a taxon as endemic if it does not occur outside Iran and as subendemic if more than 80% of its range is situated within Iran with additional occurrences only in neighbouring countries. Distribution data were taken from the Flora Iranica^37^ and the Flora of Iran^38^, supplemented by data on new species and new records published after these two floras (Table S1.2). A total of 5,970 records were geo-referenced (Fig. S1.1) with a precision of at least 0.25 × 0.25 degrees. Additionally, records on the elevational distribution have been collected for both endemic and non-endemic Asteraceae taxa, allowing the comparison of their distribution along the elevational gradient.

### Data analyses

AEs were identified using endemicity analysis, formalized by refs^40,99^ as implemented in the program NDM/VNDM 3^100^. Briefly, for each putative AE (i.e., set of grid cells) an endemicity score is calculated as the sum of the endemicity scores of each constituting species^99^. The endemicity score for each species in an AE varies between 0 (non scoring: no record inside the AE) and 1 (species found in all cells of the AE, and in no cell outside the AE); for more details see ref.^99^. Therefore, the endemicity score of an area is affected both by the number of species supporting an area and the endemicity scores of these species. One of the advantages of this approach is its ability to recognize overlapping AEs. These may be independent if defined by different sets of species^99^, and are to be expected when different environments are found in the same cell.

When analysing a dataset, NDM/VNDM converts the given geographic coordinates of a species into presence/absence data per grid cell^42^. The data were analysed with a cell size of one degree longitude and latitude (approx. 90 × 110 km in the study area) resulting in 192 cells; this is only slightly larger than the optimal grid size of 0.98 × 0.98° determined from point density data by the program (option “autogrid”). The advantage of this relatively large cell size is that the effects of sampling bias in the original point records are reduced. Heuristic searches for AEs consisted of 100 replicates, temporarily saving sets within 0.99 of the score of the set being swapped (see ref.^99^ for further details). Sets identified by this search were retained only if they had at least 10 contributing species and an endemicity score of at least 2. The threshold of 10 species was chosen empirically as the number at which delimitation of AEs became stable. Swapping was done one cell at a time and overlapping subsets were kept if 30% of the species were unique, whereas suboptimal sets were not retained. In order to reduce the level of redundancy in the inferred AEs, consensus areas were constructed^101^. To this end, we used the loose consensus rule (considered sufficiently detailed for large-scale studies)^101^, i.e., areas are added when each area shares at least 25% of its defining species with at least one, but not necessarily all, of the other areas in the consensus. The list of taxa contributing to the score of identified AEs is given in Table S1.3.

The relationship between endemic richness (i.e., number of endemic taxa) and mean elevation as well as the relationships between endemic richness and degree of endemicity (i.e., maximum endemicity score) on one hand and topographical complexity and elevational amplitude on the other hand were tested using generalized linear models with quasi-Poisson distributions and logarithmic link functions as implemented in the function *glm* of the programming environment R^102^ version 3.0.1. Topographic complexity was calculated as the ratio between the surface area, extracted from a digital elevation model with a resolution of approximately 119 × 119 meters, and the planimetric area of each grid cell^103^, using the extension DEM Surface Tools for ArcGIS^104,105^ in ArcGIS 10 (Esri, Redlands, CA, USA).

The relationship between endemism and elevation was assessed visually by plotting planimetric area against the optimal distribution of species over the elevational amplitude. To this end, we calculated the proportion of pixels of the digital elevation model within 100 meter elevational belts. Species’ elevational optima were defined as the average elevation (in meters) of all records of a given species. The relation between endemism (as the percentage of endemism) and elevation (in bins of 100 m) was tested using linear models as implemented in the function *lm* of the programming environment R.

## Electronic supplementary material

Supplementary Information
