# Supplementary material for: Hotspots within a global biodiversity hotspot - areas of endemism are associated with high mountain ranges
Source: Sci Rep. 2018 Jul 9;8:10345. doi: 10.1038/s41598-018-28504-9 (PMC6037708; doi:10.1038/s41598-018-28504-9)
Supplement: Supplementary file 1 — Supplementary Information [file 41598_2018_28504_MOESM1_ESM.docx]

SUPPORTING INFORMATION

**Hotspots within a global biodiversity hotspot - areas of endemism are associated with high mountain ranges**

Jalil Noroozi, Amir Talebi, Moslem Doostmohammadi, Sabine B. Rumpf, Hans Peter Linder, Gerald M. Schneeweiss

**Appendix S1 Supplementary Tables and Figures**

**Table S1.1** List of endemic taxa used in this study**,** their presence in each mountain range and the number of recorded localities for each taxon.

| **Taxon** | **Distribution in mountain ranges** | **Optimum elevation (m a.s.l.)** | **Number of records** |
| --- | --- | --- | --- |
| Achillea aucheri Boiss. subsp. aucheri | Alborz, NW Iran | 2999 | 5 |
| Achillea aucheri Boiss. subsp. glabra Hub.-Mor. | Alborz | 4288 | 1 |
| Achillea callichroa Boiss. | Zagros | 2909 | 1 |
| Achillea eriophora DC. | Zagros, Yazd-Kerman, Makran | 1440 | 36 |
| Achillea kellalensis Boiss. & Hausskn. | Zagros | 3093 | 1 |
| Achillea millefolium L. subsp. elbursensis Hub.-Mor. | Alborz | 3116 | 35 |
| Achillea oxyodonta Boiss. | Alborz | 1627 | 13 |
| Achillea pachycephala Rech.f. | Kopet Dagh | 1639 | 6 |
| Achillea talagonica Boiss. var. oxylepis (Boiss. & Hausskn.) Hub.-Mor. | Zagros, Yazd-Kerman | 1877 | 20 |
| Achillea talagonica Boiss. var. talagonica | Alborz, Zagros, NW Iran | 1951 | 17 |
| Amberboa sosnovskyi Iljin | NW Iran | 1540 | 1 |
| Amberboa zanjanica Ranjbar & Negaresh | NW Iran | 1417 | 1 |
| Anthemis altissima L. var. discoidea Iranshahr | Alborz | 2033 | 2 |
| Anthemis atropatana lranshahr | NW Iran | 1717 | 17 |
| Anthemis austroiranica Rech.f., Aell. & Esfand. | Lowland | 907 | 40 |
| Anthemis brachystephana Bornm. & Gauba | Lowland | 1234 | 18 |
| Anthemis bushehrica Iranshahr | Lowland | 23 | 3 |
| Anthemis fungosa Boiss. & Hausskn. | Lowland | 1 | 1 |
| Anthemis gayana Boiss. | Zagros, Yazd-Kerman | 2112 | 25 |
| Anthemis gilanica Bornm. & Gauba | Alborz, Zagros | 1416 | 19 |
| Anthemis gillettii Iranshahr | Lowland | 200 | 3 |
| Anthemis gracilis Iranshahr | Zagros | 2599 | 1 |
| Anthemis hemistephana Boiss. | Lowland | 997 | 1 |
| Anthemis leptophylla Eig. | Lowland | 67 | 6 |
| Anthemis lorestanica Iranshahr | Zagros | 1935 | 6 |
| Anthemis mazandaranica Iranshahr | Alborz, NW Iran | 1797 | 3 |
| Anthemis mirheydari Iranshahr | Lowland | 1068 | 4 |
| Anthemis moghanica Iranshahr | Lowland | 766 | 10 |
| Anthemis persica Boiss. | Lowland | 1165 | 23 |
| Anthemis schizostephana Boiss. & Hausskn. | Zagros, NW Iran | 1574 | 18 |
| Anthemis susiana Nábělek | Lowland | 64 | 16 |
| Anthemis talyschensis A. Fedor. | NW Iran | 1549 | 2 |
| Anthemis triumfettii (L.) All. subsp. decumbens Iranshahr | NW Iran | 1639 | 1 |
| Anthemis triumfettii (L). All. subsp. khorasanica (Rech.f.) lranshahr | Alborz | 2071 | 15 |
| Artemisia gypsacea Krasch., Popov & Lincz. ex Poljakov | Kopet Dagh | 2054 | 3 |
| Artemisia kermanensis Podl. | Yazd-Kerman, Makran | 2158 | 9 |
| Artemisia khorassanica Podl. | Lowland | 1367 | 19 |
| Artemisia melanolepis Boiss. | Alborz, NW Iran | 3500 | 10 |
| Artemisia splendens Willd. | Alborz, NW Iran | 2903 | 47 |
| Aster bachtiaricus Mozaff. | Zagros | 2380 | 1 |
| Atractylis delvarii Mozaff. | Lowland | 8 | 1 |
| Calendula aurantiaca Kotschy ex Boiss. | Zagros | 1566 | 1 |
| Carduus transcaspicus Gandog. subsp. macrocephalus (Arenes) Kazmi | Alborz | 1906 | 30 |
| Centaurea albonitens Turrill | NW Iran | 2015 | 10 |
| Centaurea amadanensis Sch.Bip. var. amadanensis | Zagros | 1764 | 6 |
| Centaurea amadanensis Sch.Bip. var. gymnoclada (Jaub. & Spech) Wagenitz | Zagros | 2107 | 7 |
| Centaurea amadanensis Sch.Bip. var. interrupta Heimerl | Zagros, NW Iran | 2085 | 24 |
| Centaurea aucheri (DC.) Wagenitz subsp. elbursensis Wagenitz | Alborz, NW Iran | 2394 | 16 |
| Centaurea aucheri (DC.) Wagenitz subsp. farsistanica Wagenitz | Zagros | 2151 | 10 |
| Centaurea aucheri (DC.) Wagenitz subsp. indistincta Wagenitz | Alborz, Zagros, NW Iran | 2054 | 12 |
| Centaurea aziziana Rech.f. | Alborz, NW Iran | 1593 | 10 |
| Centaurea bachtiarica Hayek & Bornm. | Zagros | 2552 | 1 |
| Centaurea balsamita Lam. subsp kermanensis (Bornm.) Wagenitz | Zagros, Yazd-Kerman | 2162 | 3 |
| Centaurea bavegehensis Ranjbar & Negaresh | Zagros | 1410 | 1 |
| Centaurea carduiformis DC. subsp. iranica Wagenitz | Alborz | 1749 | 5 |
| Centaurea congesta Wagenitz | NW Iran | 1987 | 8 |
| Centaurea elbursensis Boiss. & Buhse | Alborz, NW Iran | 2081 | 2 |
| Centaurea elymaitica Mozaff. | Lowland | 1167 | 1 |
| Centaurea gabrielae (Bornm.) Wagenitz | Yazd-Kerman, Makran | 1561 | 11 |
| Centaurea galactochora Rech.f. | Kopet Dagh | 1717 | 3 |
| Centaurea gaubae (Bornm.) Wagenitz | Zagros | 1978 | 18 |
| Centaurea geluensis Boiss. & Hausskn. | Zagros | 2447 | 1 |
| Centaurea ghahremanii Wagenitz & Esfand | Zagros | 2547 | 1 |
| Centaurea gilanica Bornm. | Alborz, Zagros, NW Iran | 1962 | 12 |
| Centaurea golestanica Akhani & Wagenitz | Alborz | 1432 | 1 |
| Centaurea hyrcanica Bornm. | Lowland | 1219 | 37 |
| Centaurea iljinii Czernjak | Lowland | 1169 | 6 |
| Centaurea imperialis Hausskn. ex Bornm. | Zagros, NW Iran | 1740 | 18 |
| Centaurea incanescens (DC.) Sch.Bip. | NW Iran | 1876 | 10 |
| Centaurea intricata Boiss. subsp. intricata | Lowland | 1079 | 17 |
| Centaurea irritans Wagenitz | Lowland | 1375 | 12 |
| Centaurea ispahanica Boiss. | Zagros, Yazd-Kerman | 2151 | 24 |
| Centaurea kamyaranensis Ranjbar & Negaresh | Lowland | 1394 | 1 |
| Centaurea kandavanensis Wagenitz | Alborz | 1969 | 8 |
| Centaurea karamianiae Negaresh | Zagros | 1464 | 1 |
| Centaurea khuzestanica Mozaff. | Lowland | 1109 | 1 |
| Centaurea koeieana Bornm. subsp. koeieana | Zagros | 1436 | 8 |
| Centaurea koeieana Bornm. subsp. multicaulis Negaresh | Lowland | 1371 | 1 |
| Centaurea lachnopus Rech.f. | Lowland | 1153 | 3 |
| Centaurea leuzeoides (Jaub. & Spech) Walp. | Alborz, Zagros, NW Iran | 1840 | 29 |
| Centaurea luristanica Rech.f. | Zagros | 1878 | 4 |
| Centaurea microlonchoides Boiss. | Yazd-Kerman, NW Iran | 1723 | 5 |
| Centaurea nemecii Nábělek | Zagros, NW Iran | 1752 | 6 |
| Centaurea ochrocephala Wagenitz | NW Iran | 2147 | 4 |
| Centaurea orumiensis Ranjjbar & Negaresh | NW Iran | 1780 | 1 |
| Centaurea pabotii Wagenitz | Lowland | 524 | 6 |
| Centaurea paradoxa Mozaff. | Lowland | 1332 | 1 |
| Centaurea persica Boiss. | Zagros, NW Iran | 2274 | 26 |
| Centaurea phaeopappa (DC.) Sch.Bip. subsp. jiroftensis Ranjbar & Negaresh | Lowland | 1209 | 1 |
| Centaurea phaeopappoides Bordizil | NW Iran | 1540 | 1 |
| Centaurea phlomoides Boiss. & Hausskn. | Lowland | 1053 | 1 |
| Centaurea procera Mozaff. | Lowland | 1050 | 1 |
| Centaurea pseudoscabiosa Boiss. & Buhse subsp. armata Wagenitz | NW Iran | 1708 | 15 |
| Centaurea pterocaula Trautv. subsp. iranica Wagenitz. | Zagros, NW Iran | 2413 | 11 |
| Centaurea ravansarensis Ranjbar & Negaresh | Lowland | 1387 | 1 |
| Centaurea regia Boiss. subsp. javanroudensis Ranjbar & Negaresh | Zagros | 1423 | 2 |
| Centaurea schmidii Wagenitz | Kopet Dagh | 2300 | 1 |
| Centaurea shahuensis Ranjbar & Negaresh | Zagros | 1426 | 1 |
| Centaurea shehbazii Ranjbar & Negaresh | Zagros | 1450 | 1 |
| Centaurea sintenisiana Gand. | Lowland | 1269 | 16 |
| Centaurea solitaria Ranjbar & Negaresh | Lowland | 1345 | 1 |
| Centaurea sosnovskyi Grossh. | Lowland | 1383 | 14 |
| Centaurea urvillei DC. subsp. deinacantha (Boiss. & Hausskn.) Wagenitz | NW Iran | 2039 | 10 |
| Centaurea ustulata DC. | Alborz, Zagros, NW Iran | 1843 | 19 |
| Centaurea wendelboi Wagenitz. | Lowland | 1113 | 2 |
| Centaurea xeranthemoides Rech.f. | Zagros | 2084 | 6 |
| Centaurea zangulensis Ranjbar & Negaresh | Alborz | 2499 | 1 |
| Centaurea zuvandica (Sosn.) Sosn. | Alborz, NW Iran | 2061 | 23 |
| Cephalorrhynchus brassicifolius (Boiss.) Tuisl | Alborz, Kopet Dagh, Zagros | 2006 | 35 |
| Cephalorrhynchus gorganicus (Rech.f. & Esfand.) Tuisl | Alborz | 1969 | 3 |
| Cephalorrhynchus kossinskyi (Krasch.) Krip. | Alborz, Kopet Dagh | 1648 | 14 |
| Cephalorrhynchus microcephalus (DC.) Schchian | Alborz, Zagros, NW Iran | 1813 | 51 |
| Chamaegeron asterellus (Bornm.) Botsch. | Yazd-Kerman | 2724 | 8 |
| Chamaegeron keredjensis (Bornm. & Gauba) Grierson | Alborz | 2558 | 1 |
| Chondrilla juncea L. var. longifolia Nasseh | Kopet Dagh | 1506 | 2 |
| Cicerbita polyclada (Boiss.) Beauverd | Zagros | 3081 | 1 |
| Cirsium bracteosum DC. var. brevicuspis Boiss. | Zagros | 1816 | 3 |
| Cirsium gadukense Petrak | Alborz | 2711 | 1 |
| Cirsium iranicum Petrak. | NW Iran | 1862 | 1 |
| Cirsium lappaceum M.Bieb. var. ferox Boiss. | Alborz, NW Iran | 2759 | 10 |
| Cirsium lappaceum M.Bieb. var. tomentosum Boiss. | Alborz, NW Iran, Zagros | 2680 | 10 |
| Cirsium pyramidale Bornm. | Yazd-Kerman | 2497 | 2 |
| Cirsium spectabile DC. | Alborz, Yazd-Kerman | 2040 | 11 |
| Cirsium strigosum (M.Bieb.) M.Bieb. var. khorassanicum Boiss. | Alborz, Kopet Dagh | 1823 | 6 |
| Cousinia adenostegia Rech.f. | Kopet Dagh | 2034 | 2 |
| Cousinia adenosticta Bornm. | Alborz | 3103 | 90 |
| Cousinia aggregata DC. | Alborz, Zagros | 1821 | 25 |
| Cousinia akredii Bornm. & Gauba | Alborz | 2353 | 5 |
| Cousinia albescens C. Winkl. & Strauss | Zagros | 2461 | 3 |
| Cousinia albida DC. | Zagros | 2450 | 2 |
| Cousinia alexeenkoana Bornm. | Zagros, Alborz, NW Iran | 2033 | 9 |
| Cousinia alfredii Bornm. & Gauba | Alborz | 2707 | 1 |
| Cousinia amicorum Tscherneva, Joharchi & F.Ghahrem. | Kopet Dagh | 2097 | 1 |
| Cousinia amplissima (Boiss.) Boiss. | Alborz, Zagros, NW Iran | 1894 | 20 |
| Cousinia antonowii C. Winkl. | Lowland | 600 | 5 |
| Cousinia arakensis Attar & Djavadi | Zagros | 2052 | 9 |
| Cousinia araneosa DC. | Zagros, Yazd-Kerman | 2336 | 30 |
| Cousinia archibaldii Rech.f. | Zagros | 2499 | 8 |
| Cousinia arctotidifolia Bunge | Kopet Dagh | 1409 | 13 |
| Cousinia ardalensis Attar & Djavadi | Zagros | 2031 | 4 |
| Cousinia argentea Mehregan & Assadi | Kopet Dagh | 1714 | 5 |
| Cousinia assadii Attar | Zagros | 2951 | 2 |
| Cousinia assyriaca Jaub. & Spech | Alborz, NW Iran | 1501 | 13 |
| Cousinia atrobracteata Attar | NW Iran | 2642 | 1 |
| Cousinia atropatana Bunge | NW Iran | 1534 | 1 |
| Cousinia attariae Assadi & Joharchi | Kopet Dagh | 1839 | 3 |
| Cousinia azerbaidjanica Djavadi, Attar & Najafi | Lowland | 1101 | 1 |
| Cousinia bachtiarica Boiss. & Hausskn. | Zagros | 2709 | 7 |
| Cousinia barbeyi C.Winkl. | Zagros | 1969 | 4 |
| Cousinia barezica Assadi | Yazd-Kerman | 2100 | 1 |
| Cousinia bazoftensis Attar | Zagros | 2513 | 3 |
| Cousinia beauverdiona Bornm. | Yazd-Kerman | 2923 | 1 |
| Cousinia belangeri DC. | Alborz, Zagros, NW Iran | 1548 | 37 |
| Cousinia bienerti Bunge | Lowland | 1379 | 3 |
| Cousinia bijarensis Rech.f. | Zagros, NW Iran | 1820 | 4 |
| Cousinia bobekii Rech.f. | NW Iran | 1562 | 20 |
| Cousinia boissieri Buhse | NW Iran | 1929 | 2 |
| Cousinia boyerahmadica Rastegar, Attar & Mirtadz. | Zagros | 1850 | 2 |
| Cousinia calcitrapa Boiss. var. calcitrapa | Zagros, Yazd-Kerman, NW Iran | 2222 | 19 |
| Cousinia calcitrapa Boiss. var. interrupta Heimerl | Zagros | 2110 | 26 |
| Cousinia calocephala Jaub. & Spech subsp. astrocephala (Hausskn. & Bornm.) Mehregan | Zagros | 2046 | 29 |
| Cousinia calocephala Jaub. & Spech subsp. behboudiana (Rech.f. & Esfand.) Mehregan | Alborz | 2033 | 45 |
| Cousinia calocephala Jaub. & Spech subsp. calocephala | Alborz, Zagros, NW Iran | 2049 | 141 |
| Cousinia calolepis Boiss. | Alborz | 2782 | 3 |
| Cousinia candolleana Jaub. & Spech | Zagros | 1829 | 2 |
| Cousinia canescens DC. | NW Iran, Zagros | 1944 | 5 |
| Cousinia cavarae Bornm. | Lowland | 1201 | 1 |
| Cousinia chaetocephala Kult. | Kopet Dagh | 1660 | 6 |
| Cousinia chamaepeuce Boiss. | Alborz | 2602 | 7 |
| Cousinia chlorocephala C. A. May. | NW Iran | 2262 | 1 |
| Cousinia chlorosphaera Bornm. | Zagros | 1534 | 16 |
| Cousinia chrysacantha Jaub. & Spech | Lowland | 1350 | 15 |
| Cousinia chrysandra Bornm. & Gauba | Kopet Dagh | 1723 | 1 |
| Cousinia commutata Bunge | Alborz | 2599 | 10 |
| Cousinia concinna Boiss. & Hausskn. | Zagros, NW Iran | 1844 | 28 |
| Cousinia concolor Bunge | Alborz, Kopet Dagh | 2065 | 2 |
| Cousinia contumax C. Winkl. & Bornm. | Zagros | 1749 | 2 |
| Cousinia cordifolia Djavadi & Attar | Alborz | 1883 | 1 |
| Cousinia crassipes Kult. | Kopet Dagh | 2170 | 1 |
| Cousinia crispa Jaub. & Spech | Alborz | 3230 | 159 |
| Cousinia curvibracteata Mehregan | Zagros | 2225 | 2 |
| Cousinia cylindracea Boiss. | Alborz, Zagros, NW Iran | 1985 | 31 |
| Cousinia cylindracea Boiss. var. patula Heimerl | Zagros, NW Iran | 1797 | 10 |
| Cousinia czerniakowskae Kult. | Kopet Dagh | 2310 | 1 |
| Cousinia dasylepis Kult. | Kopet Dagh | 1489 | 1 |
| Cousinia decipiens Boiss. & Hohen. | Alborz | 2113 | 8 |
| Cousinia decumbens Rech.f. | Alborz | 2979 | 3 |
| Cousinia denaensis Attar & Djavadi | Zagros | 2516 | 5 |
| Cousinia deserti Bunge var. longispinosa Djavadi & Attar | Kopet Dagh | 1769 | 1 |
| Cousinia diezii Rech.f. | Kopet Dagh | 1650 | 1 |
| Cousinia dipterocarpa Bornm. & Rech.f. | Kopet Dagh | 1736 | 2 |
| Cousinia discolor Bunge | Kopet Dagh | 1477 | 1 |
| Cousinia disfulensis Bornm. | Zagros | 1529 | 1 |
| Cousinia eburnea Bornm. | Zagros | 3464 | 2 |
| Cousinia edmondsonii Rech.f. | Alborz | 2044 | 2 |
| Cousinia ekbatanensis Bornm. | Zagros | 2259 | 6 |
| Cousinia elata Boiss. & Buhse | Alborz, Kopet Dagh | 1802 | 29 |
| Cousinia elburzensis Attar, Mahdigholi & Ghahr. | Alborz | 1748 | 1 |
| Cousinia erinacea Jaub. & Spach | Lowland | 1009 | 6 |
| Cousinia eriobasis Bunge | Zagros | 2020 | 9 |
| Cousinia eriophylla (Kult.) Bornm. | Kopet Dagh | 1443 | 2 |
| Cousinia eriorhiza Bornm. | Zagros | 2577 | 1 |
| Cousinia eryngioides Boiss. | Alborz, Yazd-Kerman, Kopet Dagh, NW Iran | 1784 | 29 |
| Cousinia esfandiarii Rech.f. & Aell. | Alborz | 1833 | 9 |
| Cousinia euchlora Bornm. & Rech.f. | Kopet Dagh | 1960 | 1 |
| Cousinia fabrorum Rech.f. | Alborz, Kopet Dagh | 1613 | 12 |
| Cousinia falcinella Bornm. | Lowland | 1289 | 1 |
| Cousinia firuzkuhensis Rech.f. | Alborz | 2360 | 2 |
| Cousinia fragilis C. Winkl. & Bornm. | Yazd-Kerman | 4086 | 2 |
| Cousinia freynii Bornm. | Lowland | 1365 | 4 |
| Cousinia gabrielae Bornm. | Kopet Dagh, Makran | 1740 | 3 |
| Cousinia gaharensis Attar & Djavadi | Zagros | 2292 | 1 |
| Cousinia gatchsaranica Mehregan, Assadi & Attar | Zagros | 2600 | 2 |
| Cousinia gaubae Bornm. | Alborz | 1864 | 6 |
| Cousinia gedrosiaca Bornm. & Gauba | Yazd-Kerman, Makran | 1995 | 4 |
| Cousinia ghahremanii Mirtadz. & Attar | Yazd-Kerman | 2593 | 1 |
| Cousinia gilanica Bornm. | Alborz, NW Iran | 2023 | 2 |
| Cousinia gilliatii Rech.f. | NW Iran | 1752 | 3 |
| Cousinia glaucopsis Bornm. & Rech.f. | Alborz | 1870 | 3 |
| Cousinia gmelini C. Winkl. | Alborz, NW Iran | 2416 | 4 |
| Cousinia golestanica Attar | Kopet Dagh | 1544 | 2 |
| Cousinia gracilis Boiss. | Zagros | 2262 | 8 |
| Cousinia grandis C. A. Mey. | NW Iran | 1796 | 59 |
| Cousinia hablitzlii C. A. Mey. | Alborz | 2299 | 1 |
| Cousinia hamosa C. A. Mey. | NW Iran | 1401 | 2 |
| Cousinia harazensis Rech.f. | Alborz | 2399 | 5 |
| Cousinia heliantha Bunge | Kopet Dagh | 1596 | 5 |
| Cousinia hergtiana Bornm. | Zagros | 2157 | 3 |
| Cousinia hololeuca Bunge | Lowland | 1224 | 1 |
| Cousinia horrida Kult. | Kopet Dagh | 2012 | 1 |
| Cousinia hypochionea Bornm. | Lowland | 394 | 2 |
| Cousinia hypoleuca Boiss. | Alborz, Zagros, Kopet Dagh | 2362 | 13 |
| Cousinia hypopolia Bornm.& Sint. | Lowland | 1093 | 10 |
| Cousinia ilicifolia Jaub. & Spech | Zagros | 2307 | 1 |
| Cousinia inflata Boiss. & Hausskn. | Zagros | 1495 | 1 |
| Cousinia iranica C. Winkl. & Strauss. | Zagros | 2299 | 2 |
| Cousinia iranshahriana Attar & Maroofi | Zagros | 2312 | 4 |
| Cousinia iranshahrii Rech.f. | Lowland | 1282 | 1 |
| Cousinia irritans Rech.f. | Alborz, Kopet Dagh | 2577 | 11 |
| Cousinia isfahanica Assadi | Zagros | 2155 | 1 |
| Cousinia Jaeobsii Rech.f. | Lowland | 1255 | 2 |
| Cousinia kadereitii Mehregan & Assadi | Kopet Dagh | 2236 | 2 |
| Cousinia kandavanensis Attar | Alborz | 1995 | 2 |
| Cousinia karkasensis Mehregan & Djavadi | Zagros | 2609 | 3 |
| Cousinia kashanensis Rech.f. & Esfand. | Zagros | 2077 | 1 |
| Cousinia keredjensis Bornm. & Gauba | Alborz | 1887 | 4 |
| Cousinia khorasanica Djavadi & Attar | Lowland | 1219 | 1 |
| Cousinia kilouyensis Djavadi & Attar | Zagros | 2225 | 1 |
| Cousinia komarowii (O. Kuntze) C. Winkl. | Kopet Dagh | 1863 | 4 |
| Cousinia komidjanensis Mehregan | Zagros | 2468 | 1 |
| Cousinia kornhuberi Heimerl | Zagros | 2022 | 4 |
| Cousinia kotschyi Boiss. subsp. kotschyi | Zagros, Kopet Dagh, Yazd-Kerman, NW Iran | 2091 | 65 |
| Cousinia kotschyi Boiss. subsp. khansarica (Attar & Ghahreman) Mehregan | Zagros | 2284 | 30 |
| Cousinia kurdistanica Attar | Lowland | 1307 | 1 |
| Cousinia lactiflora Rech.f. | Zagros | 3087 | 1 |
| Cousinia lasiandra Bunge | Lowland | 1124 | 7 |
| Cousinia lepida Bunge ex Boiss. | Lowland | 1195 | 7 |
| Cousinia lignosissima Rech.f. | Lowland | 1280 | 1 |
| Cousinia linczewskii Juz. | Lowland | 602 | 4 |
| Cousinia longibracteata Attar & Mirtadz. | Kopet Dagh | 1568 | 1 |
| Cousinia longifolia C. Winkl. & Bornm. | Zagros, Yazd-Kerman | 3077 | 12 |
| cousinia lordeganensis Mehregan | Zagros | 1950 | 3 |
| Cousinia lucida DC. | Alborz, Zagros | 2192 | 2 |
| Cousinia lurestanica Attar & Djavadi | Zagros | 1997 | 1 |
| Cousinia lurorum (Bornm.) Bornm. | Zagros | 2031 | 25 |
| Cousinia maassoumii Assadi | Zagros | 1839 | 2 |
| Cousinia macrocephala C. A. Mey. | NW Iran | 1560 | 22 |
| Cousinia manoumehrii Rech.f. & Esfand. | Zagros | 1842 | 1 |
| Cousinia mehreganii Assadi | Kopet Dagh | 1892 | 2 |
| Cousinia meluarmanica Rech.f. | Alborz | 1904 | 4 |
| Cousinia meshhedensis Bornm. & Rech.f. | Alborz, Kopet Dagh | 1746 | 9 |
| Cousinia microcephala C. A. Mey. | NW Iran | 1500 | 6 |
| Cousinia monocephala Bunge | Lowland | 606 | 3 |
| Cousinia mozdouranensis Djavadi & Attar | Lowland | 818 | 1 |
| Cousinia mutehensis Rech.f. | Zagros | 1880 | 1 |
| Cousinia nekarmanica Rech.f. | Alborz | 1977 | 3 |
| Cousinia neurocentra Bunge | Alborz, Yazd-Kerman | 1696 | 7 |
| Cousinia noeana Boiss. | Zagros | 1800 | 12 |
| Cousinia nujianensis Attar, Ghahr., Saber & Zarre | Zagros | 1770 | 1 |
| Cousinia odontolepis Sch.Bip. ex DC. subsp. odontolepis | Alborz, NW Iran | 1984 | 7 |
| Cousinia oligocephala Boiss. | Zagros | 2378 | 2 |
| Cousinia onopordioides Ledeb. | Alborz, Kopet Dagh, Yazd-Kerman, Makran | 1922 | 55 |
| Cousinia oreodoxa Bornm. & Sint. | Kopet Dagh | 1514 | 5 |
| Cousinia orthoclada Hausskn. & Bornm. | Zagros | 2455 | 12 |
| Cousinia oshtorankuhensis Attar | Zagros | 2977 | 2 |
| Cousinia ottonis Bornm. | Zagros | 2890 | 2 |
| Cousinia papillosa Djavadi & Attar | Kopet Dagh | 1747 | 1 |
| Cousinia parsina Jaub. & Spech. | Alborz, Zagros, NW Iran | 1706 | 4 |
| Cousinia pasargardensis Attar | Zagros | 2125 | 1 |
| Cousinia pergamacea Boiss. & Hausskn. subsp. pergamacea | Zagros, NW Iran | 1777 | 47 |
| Cousinia pergamacea Boiss. & Hausskn. subsp. sardashtensis (Rech.f.) Mehregan | NW Iran | 1530 | 2 |
| Cousinia persica Djavadi & Attar | Alborz | 1971 | 1 |
| Cousinia perspolitana Attar & Ghahr. | Zagros | 2210 | 3 |
| Cousinia pinarocephala Boiss. | Alborz, NW Iran | 2297 | 23 |
| Cousinia piptocephala Bunge | Alborz, Zagros, Kopet Dagh, Yazd-Kerman | 1739 | 18 |
| Cousinia platyacantha Bunge | Kopet Dagh | 2057 | 3 |
| Cousinia platyraphis Kult. | Kopet Dagh | 1502 | 1 |
| Cousinia pseudocandolleana Assadi | Kopet Dagh | 1601 | 1 |
| Cousinia pterocaulos (C. A. Mey.) Rech.f. | Alborz, Zagros, NW Iran | 2315 | 9 |
| Cousinia pugionifera Jaub. & Spech | Zagros | 2034 | 5 |
| Cousinia qarehbilensis Rech.f. | Lowland | 1286 | 1 |
| Cousinia raphiocephala Rech f. | Zagros | 1887 | 4 |
| Cousinia raphiostegia Rech.f. | Lowland | 1124 | 1 |
| Cousinia rechingerae Bornm. | Lowland | 768 | 7 |
| Cousinia rechingerorum Bornm. | Kopet Dagh | 1739 | 1 |
| Cousinia recurvata DC. | Alborz, Zagros, Kopet Dagh | 2356 | 8 |
| Cousinia remingerorum Bornm. | Kopet Dagh | 1502 | 1 |
| Cousinia renominata Rech.f. | Lowland | 763 | 1 |
| Cousinia rhabdodes Bornm. & Rech.f. | Alborz | 1767 | 1 |
| Cousinia rhaphiocephala Rech.f. | Zagros | 2003 | 1 |
| Cousinia sabalanica Attar, Ghahreman & Assadi | NW Iran | 1403 | 1 |
| Cousinia sabzavarensis Rech.f. | Zagros, Kopet Dagh | 1618 | 2 |
| Cousinia sagittata C.Winkl. & Strauss subsp. iranica (C. Winkl. & Strauss) Mehregan | Zagros | 2021 | 29 |
| Cousinia sagittata C.Winkl. & Strauss subsp. sagittata | Zagros | 1931 | 52 |
| Cousinia sahandica Attar & Djavadi | NW Iran | 1717 | 2 |
| Cousinia sakawensis Boiss. & Hausskn. | Zagros | 2567 | 1 |
| Cousinia saloukensis Mehregan | Kopet Dagh | 2546 | 1 |
| Cousinia sardashtensis Rech.f. | Zagros | 1534 | 1 |
| Cousinia sarzehensis Attar, Ghahreman & Assadi | Yazd-Kerman | 1686 | 2 |
| Cousinia schindleriana Bornm. & Gauba | Kopet Dagh | 2035 | 1 |
| Cousinia schiraziana Attar | Zagros | 2153 | 1 |
| Cousinia seiditzii Bunge | NW Iran | 1560 | 10 |
| Cousinia shahuensis Attar | Zagros | 1477 | 1 |
| Cousinia shahvarica Rech.f. | Alborz | 2955 | 16 |
| Cousinia sheidaii Attar, Ghahr. & Mahdigholi | Zagros | 2450 | 2 |
| Cousinia sicigera C. Winkl. & Bornm. | Yazd-Kerman | 3473 | 8 |
| Cousinia silvanica Attar | NW Iran | 2553 | 1 |
| Cousinia silyboides Jaub. & Spach subsp. disfulensis (Bornm.) Mehregan | Zagros | 1489 | 3 |
| Cousinia silyboides Jaub. & Spach subsp. silyboides | Zagros | 2414 | 6 |
| Cousinia silyboides Jaub. & Spach subsp. zardkuhensis (Attar & Ghahreman) Mehregan | Zagros | 2176 | 6 |
| Cousinia smirnowii Trautv | Alborz, Kopet Dagh | 1930 | 16 |
| Cousinia sphaerocephala Jaub. & Spech | Alborz | 2805 | 10 |
| Cousinia stahbiana Bornm. & Gauba | Kopet Dagh | 1483 | 11 |
| Cousinia strausii Hausskn. & Winkl. ex Winkl. | Zagros | 2436 | 2 |
| Cousinia subinflata Bornm. | Zagros | 3064 | 1 |
| Cousinia subpectinata Mirtadz., Attar & Assadi | Yazd-Kerman | 2641 | 1 |
| Cousinia tabriziana Bunge | NW Iran | 1637 | 10 |
| Cousinia taybadensis Djavadi & Attar | Kopet Dagh | 1478 | 1 |
| Cousinia tenuifolia C. A. Mey. | NW Iran | 1596 | 11 |
| Cousinia tenuiramula Rech.f. | Zagros | 2460 | 2 |
| Cousinia termei Rech.f. | Lowland | 1304 | 1 |
| Cousinia tetanocephala Bornm. & Gauba | Kopet Dagh | 1812 | 1 |
| Cousinia thamnodes Borss. & Hausskn. | Zagros | 2277 | 3 |
| Cousinia touchalensis Attar | Alborz | 1929 | 1 |
| Cousinia trachylepis Bunge | Alborz | 1463 | 9 |
| Cousinia trachyphyllaria Bornm. & Rech. f . | Kopet Dagh | 1566 | 4 |
| Cousinia turcomanica C. Winkl. | Alborz, Kopet Dagh | 1517 | 9 |
| Cousinia urumiensis Bornm. | NW Iran | 1726 | 17 |
| Cousinia verbascifolia Bunge | Lowland | 1195 | 40 |
| Cousinia wendelboi Rech.f. | Alborz | 2073 | 1 |
| Cousinia wilhelminae Rech.f. | NW Iran | 1849 | 3 |
| Cousinia xiphiolepis Boiss. | Alborz | 2645 | 7 |
| Cousinia yasujensis Attar | Zagros | 2231 | 1 |
| Crepis alfredii Bornm. | Lowland | 358 | 1 |
| Crepis armena DC. subsp. longibracteata Babcock | NW Iran | 2289 | 1 |
| Crepis asadbarensis Bornm. ex Rech.f. | Alborz | 3004 | 14 |
| Crepis ciliata C. Koch | Alborz | 2250 | 2 |
| Crepis connexa Babc. | Zagros | 3096 | 1 |
| Crepis demavendi Bornm. | Alborz | 2772 | 5 |
| Crepis elbursensis Boiss. | Alborz, NW Iran | 2982 | 11 |
| Crepis elymaitica Bornm. | Zagros | 2985 | 4 |
| Crepis gaubae Bornm. | Alborz | 1915 | 3 |
| Crepis heterotricha DC. subsp. heterotricha | Zagros, Yazd-Kerman | 3571 | 6 |
| Crepis heterotricha DC. subsp. lobata Babcock | Alborz | 3654 | 12 |
| Crepis khorassanica Boiss. | Kopet Dagh | 1754 | 2 |
| Crepis papposissima Babcock | Alborz | 2904 | 1 |
| Crepis quercifolia Bornm. & Gauba | Alborz, Zagros, NW Iran | 1619 | 7 |
| Crepis sahendi Boiss. & Buhse | NW Iran | 3059 | 22 |
| Crepis semnanensis N. Heidarnia & M. Assadi, | Lowland | 1281 | 1 |
| Crepis straussii Bornm. | Zagros | 1700 | 8 |
| Crepis willemetioides Boiss. | Alborz | 1693 | 15 |
| Cyanus ouramanicus Ranjbar & Negaresh | Zagros | 1411 | 1 |
| Cyanus persicus Ranjbar & Negaresh | Kopet Dagh | 1834 | 1 |
| Cyanus tabrizianus Ranjbar & Negaresh | NW Iran | 2014 | 1 |
| Dolichorrhiza persica (Boiss.) B. Nord. | Alborz, NW Iran | 2873 | 2 |
| Doronicum bracteatum Edmondson | Zagros, NW Iran | 2614 | 6 |
| Doronicum wendelboi Edmondson | Alborz | 1871 | 4 |
| Echinops abazariae Mozaff. | Lowland | 990 | 3 |
| Echinops arachniolepis Rech.f. | Kopet Dagh | 2064 | 2 |
| Echinops aucheri Boiss. | Yazd-Kerman | 1809 | 3 |
| Echinops austro-iranicus Mozaff. | Lowland | 1075 | 14 |
| Echinops avajensis Mozaff. | Zagros, NW Iran | 2049 | 5 |
| Echinops bakhtiaricus Rech.f. | Lowland | 843 | 4 |
| Echinops cephalotes DC. | Alborz, Zagros | 1466 | 38 |
| Echinops ceratophorus Boiss. | Zagros, Yazd-Kerman | 1995 | 17 |
| Echinops cervicermis Bornm. | Yazd-Kerman | 2328 | 1 |
| Echinops chorassanicus Bunge | Lowland | 1367 | 32 |
| Echinops cyanocephalus Boiss. | Zagros | 2251 | 2 |
| Echinops delicatus Mozaff. | Lowland | 359 | 1 |
| Echinops dichrous Boiss. & Hausskh. | Lowland | 651 | 10 |
| Echinops disfulensis Bornm. | Lowland | 820 | 1 |
| Echinops ecbatanus Bornm. | Zagros, NW Iran | 1795 | 7 |
| Echinops ecbatul Bornm. ex Rech.f. | Alborz, Zagros, NW Iran | 1791 | 16 |
| Echinops elbursensis Rech.f. | Alborz | 2582 | 12 |
| Echinops elymaitica Bornm. | Zagros | 2290 | 19 |
| Echinops endotrichus Rech.f. | Zagros | 1586 | 7 |
| Echinops erioceras Bornm. | Zagros | 2187 | 5 |
| Echinops eriophorus Bornm. | Lowland | 847 | 1 |
| Echinops farsicus Rech.f. | Zagros | 1708 | 12 |
| Echinops gedrosiacus Bornm. var. glaber Mozaff. | Lowland | 1090 | 4 |
| Echinops glanduloso-punctatus Rech.f. | Lowland | 1266 | 2 |
| Echinops haussknechtii Boiss. | Zagros, NW Iran | 1540 | 12 |
| Echinops hebelepis DC. | Zagros | 2237 | 2 |
| Echinops heteromorphus Bunge | Kopet Dagh | 1649 | 6 |
| Echinops ilicifolius Bunge | Yazd-Kerman | 1540 | 7 |
| Echinops iranshahrii Rech.f. | Zagros | 1876 | 5 |
| Echinops jesdianus Boiss. | Yazd-Kerman | 2302 | 5 |
| Echinops kazerunensis Mozaff. | Lowland | 845 | 2 |
| Echinops keredjensis Rech.f. | Alborz, Zagros | 1659 | 20 |
| Echinops kermanshahanicus Mozaff. var. papillosus (Rech.f.) Mozaff. | Lowland | 563 | 4 |
| Echinops khansaricus Mozaff. | Zagros | 2223 | 8 |
| Echinops khuzistanicus Mozaff. | Lowland | 399 | 4 |
| Echinops koelzii Rech.f. | Alborz, NW Iran | 1903 | 20 |
| Echinops kordicus Boiss. & Hausskh. | Zagros | 2460 | 1 |
| Echinops kotschyi Boiss. | Zagros | 2688 | 4 |
| Echinops lalesarensis Bornm. | Yazd-Kerman, Kopet Dagh | 2255 | 6 |
| Echinops laricus Mozaff. | Lowland | 882 | 3 |
| Echinops lasiolepis Bunge | Lowland | 1387 | 5 |
| Echinops leiopolyceroides Mozaff. | Alborz, Kopet Dagh | 1525 | 12 |
| Echinops longipenicillatus Mozaff. & Ghahr. | Lowland | 757 | 14 |
| Echinops macrophyllus Boiss. & Hausskn. var. papillosus Mozaff. | Alborz, Zagros | 2019 | 15 |
| Echinops macrophyllus Boiss. & Hausskn. var. macrophyllus | Lowland | 1009 | 9 |
| Echinops macrophyllus Boiss. & Hausskn. var. laciniatus Mozaff. | Zagros | 1928 | 29 |
| Echinops mosulensis Rech.f. var. papillosus Mozaff. | Alborz, Zagros | 1695 | 40 |
| Echinops nizvanus Rech.f. | Alborz | 2148 | 6 |
| Echinops pabotii Rech.f. | Yazd-Kerman | 1737 | 1 |
| Echinops pachyphyllus Rech.f. | Lowland | 576 | 1 |
| Echinops persepolitanus Rech.f. | Zagros | 1837 | 10 |
| Echinops polychromus Rech.f. | Lowland | 241 | 4 |
| Echinops polygamus Bunge | Alborz, Zagros | 1634 | 4 |
| Echinops procerus Mozaff. | Kopet Dagh | 1648 | 3 |
| Echinops psammophilus Mozaff. | Lowland | 182 | 5 |
| Echinops quercetorum Mozaff. | Zagros | 1626 | 1 |
| Echinops robustus Bunge | Lowland | 1269 | 13 |
| Echinops sabzevarensis Mozaff. | Alborz, Zagros, Kopet Dagh | 1545 | 5 |
| Echinops shahrudensis Mozaff. & Ghahreman | Alborz | 1703 | 2 |
| Echinops shulabadensis Mozaff. | Alborz, Zagros | 1595 | 3 |
| Echinops sojakii Rech.f. | Zagros | 1791 | 5 |
| Echinops taftanicus Mozaff. | Makran | 1975 | 1 |
| Echinops tenuistus Rech.f. | Zagros, Yazd-Kerman | 1582 | 9 |
| Echinops viscidulus Mozaff. | Zagros | 2027 | 16 |
| Erigeron acris L. subsp. arctophilus (Rech.f.) Rech.f. | Alborz, NW Iran, Kopet Dagh | 2491 | 10 |
| Erigeron acris L. subsp. asadbarensis (Vierh.) Rech.f. | Alborz, Zagros, NW Iran | 2364 | 16 |
| Erigeron acris L. subsp. lalehzaricus Rech.f. | Yazd-Kerman | 3870 | 4 |
| Erigeron hyrcanicus Bornm. & Vierh. | Alborz | 3236 | 14 |
| Erigeron uniflorus L. subsp. daënensis (vierh.) Rech.f. | Zagros | 3757 | 2 |
| Erigeron uniflorus L. subsp. elbursensis (Boiss.) Rech.f. | Alborz | 3638 | 34 |
| Grantia arachnoidea Boiss. | Lowland | 81 | 4 |
| Grantia discoidea Bunge ex Boiss. | Yazd-Kerman, Makran | 1980 | 3 |
| Helichrysum artemisioides Boiis. & Hausskn. | Zagros | 1962 | 6 |
| Helichrysum athanaton Georgiadou & Rech.f. | Zagros | 2612 | 3 |
| Helichrysum davisianum Rech.f. | Yazd-Kerman | 3051 | 4 |
| Helichrysum globiferum Boiss. | Alborz, NW Iran, Zagros | 1576 | 31 |
| Helichrysum leucocephalum Boiss. | Zagros, Yazd-Kerman, Makran | 1515 | 27 |
| Helichrysum makranicum (Rech.f. & Esfand.) Rech.f. | Lowland | 935 | 1 |
| Helichrysum oligocephalum DC. | Alborz, Zagros, NW Iran | 2379 | 65 |
| Helichrysum oocephalum Boiss. | Alborz, Zagros, Kopet Dagh | 1611 | 12 |
| Helichrysum persicum F.Ghahrem. & Noori | Kopet Dagh | 1734 | 1 |
| Helichrysum psychrophilum Boiss. | Alborz, NW Iran | 3463 | 203 |
| Hertia angustifolia (DC.) O. Kuntze | Alborz, Zagros, Yazd-Kerman | 1877 | 42 |
| Heteroderis pusilla Boiss. var. khorassanica Nasseh | Lowland | 1149 | 5 |
| Hieracium azerbaijanense Lack | NW Iran | 1612 | 3 |
| Hieracium cheirifolium Boiss. & Hausskn. | Zagros | 2600 | 5 |
| Hieracium piranshahricum Tavakkoli & Assadi | Lowland | 1349 | 2 |
| Hymenocephalus rigidus Jaub. & Spech | Zagros | 1931 | 2 |
| Inula persica F.Ghahrem. & Narimisa | Yazd-Kerman | 2771 | 1 |
| Inula rajamandii Narimisa & F.Ghahrem. | NW Iran | 1732 | 1 |
| Iranecio elbrusensis (Boiss.) B. Nord. | Alborz | 2635 | 18 |
| Iranecio oligolepis (Boiss.) B. Nord. | Alborz | 3852 | 4 |
| Iranecio paucilobus (DC.) B. Nord. | Alborz, Zagros, NW Iran | 2585 | 51 |
| Jurinea bungei Boiss. | Zagros, Yazd-Kerman | 1912 | 5 |
| Jurinea cartilaginea Mozaff. | Lowland | 490 | 1 |
| Jurinea catharinae Iljin. | Lowland | 1270 | 6 |
| Jurinea cordata Boiss. & Hausskn. | Zagros | 1691 | 2 |
| Jurinea eriobasis DC. | Zagros | 2200 | 9 |
| Jurinea gabrielae Bornm. | Kopet Dagh | 1873 | 1 |
| Jurinea gedrosiaca Bornm. | Lowland | 760 | 2 |
| Jurinea giviensis Mirtadz. | Alborz | 1496 | 1 |
| Jurinea heterophylla (Jaub. & Spech) Borss. | Alborz, Zagros, NW Iran | 1778 | 7 |
| Jurinea inuloides Boiss. & Hausskn. | Alborz | 3096 | 1 |
| Jurinea jedresiaca Bornm. | Lowland | 600 | 1 |
| Jurinea kepetensis Rech.f. | Kopet Dagh | 1728 | 2 |
| Jurinea leptoloba DC. | NW Iran | 1712 | 7 |
| Jurinea macrocephala DC. subsp. elbursensis Wagenitz. | Alborz | 1899 | 3 |
| Jurinea meda Bornm. | Zagros | 2877 | 26 |
| Jurinea mobayenii Ghahreman & Mirtadzadini | Yazd-Kerman | 2585 | 1 |
| Jurinea monocephala Aitch. & Hemsl. subsp. monocepahala | Kopet Dagh | 1907 | 1 |
| Jurinea multicaulis DC. | NW Iran | 1593 | 3 |
| Jurinea prasinophylla Rech.f. | Zagros | 2411 | 2 |
| Jurinea proteoides Boiss & Hausskn. | Zagros | 1845 | 1 |
| Jurinea radians Boiss. subsp. radians | Alborz, Kopet Dagh, NW Iran | 1414 | 20 |
| Jurinea sharifiana Rech.f. | Alborz | 1855 | 2 |
| Jurinea stenocalathia Rech.f. | Alborz, Yazd-Kerman, Kopet Dagh | 1563 | 6 |
| Jurinea viciosoi Pau | Zagros | 2551 | 2 |
| Jurinella frigida (Boiss.) Wagenitz | Alborz | 3479 | 52 |
| Jurinella microcephala (Boiss.) Wagenitz | Alborz, Kopet Dagh | 2465 | 20 |
| Jurinella moschus (Habl.) Bobrov. | NW Iran | 3052 | 41 |
| Karvandarina aphylla Rech.f. | Lowland | 1133 | 9 |
| Klasea nana Ranjbar & Negaresh | Kopet Dagh | 1480 | 1 |
| Klasea sanandajensis Ranjbar & Negaresh | Zagros | 1939 | 1 |
| Lactuca azerbaijanica Rech.f. | Alborz, NW Iran | 1447 | 4 |
| Lactuca denaensis N. Kilian & Djavadi | Zagros | 3714 | 1 |
| Lactuca hazaranensis Djavadi & N. Kilian | Yazd-Kerman | 2899 | 1 |
| Lactuca polyclada Boiss. | Zagros | 3162 | 2 |
| Launaea acanthodes (Boiss.) O. Kuntze | Lowland | 1323 | 124 |
| Launaea bornmuelleri (Hausskn. ex Bornm.) Bornm. | Lowland | 86 | 1 |
| Launaea peistocarpa (Boiss.) Rech.f. | Kopet Dagh | 2178 | 4 |
| Leontodon hispidus L. var. mazanderanicus Rech.f. | Alborz, NW Iran | 1647 | 15 |
| Leontodon stenocalathius Rech.f. | Alborz | 2285 | 2 |
| Ligularia persica Boiss. | Alborz | 2876 | 17 |
| Myopordon aucheri Boiss. | Zagros | 3354 | 2 |
| Myopordon damavandica Mozaff. | Alborz | 3741 | 1 |
| Myopordon hyrcanum (Bornm.) Wagenitz | Alborz | 3233 | 4 |
| Myopordon persicum Boiss. | Zagros | 3380 | 4 |
| Onopordon caramanicum (Bornm.) Bornm. | Yazd-Kerman, Makran, Kopet Dagh | 1779 | 13 |
| Pentanema flexuosum (Boiss. & Hausskn.) Rech.f. | Zagros, NW Iran | 1743 | 7 |
| Pentanema multicaule Boiss. | Zagros | 2048 | 6 |
| Pentanema pulicariiforme (DC.) Rech.f. | Alborz, Zagros | 2226 | 16 |
| Phagnalon persicum Boiss. | Zagros, Yazd-Kerman | 2879 | 13 |
| Picris strigosa M.Bieb. subsp. gonicaula (Boiss.) Lack | Zagros, Yazd-Kerman, Alborz | 2095 | 24 |
| Platychaete aucheri (Boiss.) Boiss. | Lowland | 886 | 22 |
| Platychaete mucronifolia (Boiss.) Boiss. | Lowland | 430 | 10 |
| Platychaete velutina Boiss. & Hausskn. | Lowland | 470 | 3 |
| Postia bombycina Boiss. & Hausskn. | Lowland | 737 | 4 |
| Postia puberula Boiss. & Hausskn. | Lowland | 1068 | 11 |
| Psephellus congestus (Wagenitz) Wagenitz | NW Iran | 1734 | 3 |
| Psephellus khalkhalensis Ranjbar & Negaresh | NW Iran | 2113 | 3 |
| Psychrogeton aellenii (Rech.f.) Grierson | Alborz, Kopet Dagh | 3343 | 2 |
| Psychrogeton chionophilus (Boiss.) Krasch. | Zagros | 3625 | 1 |
| Psychrogeton persicus (Boiss.) Grierson | Zagros, Yazd-Kerman, Kopet Dagh | 2762 | 16 |
| Rhaponticoides bachtiarica (Boiss. & Hausskn.) L. Martins | Zagros | 1824 | 1 |
| Rhaponticum insigne (Boiss.) Wagenitz | NW Iran | 1592 | 6 |
| Sclerorhachis leptoclada Rech.f. | Kopet Dagh | 2020 | 2 |
| Sclerorhachis platyrachis (Boiss.) Podl. ex Rech.f. | Lowland | 1326 | 30 |
| Scorzonera calyculata Boiss. | Alborz, Zagros, NW Iran | 1997 | 92 |
| Scorzonera flaccida Rech.f. | Zagros | 2454 | 3 |
| Scorzonera grossheimii Lipsch. & Vassilcz | Alborz, Zagros, NW Iran | 2190 | 7 |
| Scorzonera helodes Rech.f. | Zagros | 2263 | 2 |
| Scorzonera intricata Boiss. | Zagros, Yazd-Kerman, Makran | 2626 | 22 |
| Scorzonera ispahanica Boiss. | Zagros | 2067 | 6 |
| Scorzonera joharchii S.R. Safavi | Lowland | 1367 | 2 |
| Scorzonera kandavanica Rech.f. | Alborz | 2901 | 7 |
| Scorzonera luristanica Rech.f. var. lanata Safavi | Zagros | 1416 | 2 |
| Scorzonera luristanica Rech.f. var. luristanica | Zagros, NW Iran | 1869 | 33 |
| Scorzonera microcalathia (Rech.f.) Rech.f. | Yazd-Kerman, Kopet Dagh, Makran | 1547 | 11 |
| Scorzonera mucida Rech.f., Aell. & Esfand. | Alborz, Zagros, Yazd-Kerman, Kopet Dagh, Makran | 1675 | 64 |
| Scorzonera nivalis Boiss. & Hausskn. | Zagros | 3289 | 1 |
| Scorzonera persica Boiss. & Buhse | Alborz, NW Iran | 2582 | 2 |
| Scorzonera perspolitana Boiss. | Zagros | 1976 | 14 |
| Scorzonera psychrophila Boiss. & Hausskn. | Zagros | 3052 | 3 |
| Scorzonera renzii Rech.f. | NW Iran | 1861 | 6 |
| Scorzonera rupicola Hausskn. | Zagros, NW Iran | 2275 | 18 |
| Scorzonera seidlitzii Boiss. | NW Iran | 2309 | 1 |
| Scorzonera stenocephala Boiss. | Alborz, Zagros | 2922 | 15 |
| Scorzonera subaphylla Boiss. | Zagros | 3188 | 6 |
| Scorzonera szovitsii DC. | NW Iran | 1618 | 11 |
| Scorzonera wendelboi Rech.f. | Alborz | 2507 | 3 |
| Scorzonera xylobasis Rech.f. | Alborz | 2793 | 1 |
| Senecio eligulatus B. Nord., Moussavi & Djavadi | Yazd-Kerman | 3486 | 1 |
| Senecio iranicus B. Nord. | Alborz | 4107 | 5 |
| Senecio joharchii F. Ghahrem., Ezazi, Rahch. & Attar | Kopet Dagh | 1932 | 1 |
| Senecio kotschyanus Boiss. | Zagros | 3914 | 1 |
| Senecio lipskyi Lomak. | NW Iran | 2140 | 6 |
| Senecio subnivalis Ajani, Noroozi & Nord. | Yazd-Kerman | 3880 | 2 |
| Senecio vulcanicus Boiss. | Alborz | 3831 | 5 |
| Serratula bachtiarica Boiss. & Hausskn. | Zagros | 1852 | 1 |
| Serratula calcarea Mozaff. | NW Iran | 1412 | 1 |
| Serratula gracillima Rech.f. | Lowland | 322 | 1 |
| Serratula grandifolia P. H. Davis | Zagros | 2241 | 3 |
| Serratula haussknechtii Boiss. | Alborz, Zagros, NW Iran | 2340 | 9 |
| Serratula melanocheila Boiss. & Hausskn. | Zagros | 3211 | 2 |
| Serratula suffulta Rech.f. | Zagros | 2319 | 1 |
| Serratula viciifolia Boiss. & Hausskn. | Zagros, NW Iran | 2490 | 3 |
| Tanacetum archibaldii Podl. | Alborz | 3363 | 1 |
| Tanacetum bachtiaricum Mozaff. | Zagros | 2661 | 3 |
| Tanacetum budjnurdense (Rech.f.) Tzvel. | Kopet Dagh | 1494 | 1 |
| Tanacetum dumosum Boiss. | Zagros | 2966 | 16 |
| Tanacetum elbursense Mozaff. | Alborz | 1898 | 1 |
| Tanacetum hololeucum (Bornm.) Podl. | Alborz | 2890 | 23 |
| Tanacetum joharchii Sonboli & Kaz.Osaloo | Kopet Dagh | 2001 | 1 |
| Tanacetum khorassanicum (Krasch.) Parsa | Kopet Dagh | 1912 | 6 |
| Tanacetum lingulatum (Boiss.) Bornm. | Alborz, Zagros, Yazd-Kerman | 1871 | 18 |
| Tanacetum paradoxum Bornm. | Zagros | 1619 | 3 |
| Tanacetum persicum (Boiss.) Mozaff. | Alborz, Zagros, Kopet Dagh, NW Iran | 2524 | 34 |
| Tanacetum polycephalum Sch.Bip. subsp. azerbaidjanicum Podl. | Zagros, NW Iran | 1610 | 18 |
| Tanacetum polycephalum Sch.Bip. subsp. farsicum Podl. | Zagros | 2356 | 33 |
| Tanacetum polycephalum Sch.Bip. subsp. junesarense (Bornm.) Podl. | Alborz | 2947 | 1 |
| Tanacetum salsugineum Podl. | NW Iran | 1647 | 1 |
| Tanacetum stapfianum (Rech.f.) Podl. | Zagros | 1633 | 3 |
| Tanacetum tarighii Sonboli | NW Iran | 1762 | 2 |
| Tanacetum tenuisectum (Boiss.) Podl. | Alborz | 2432 | 22 |
| Tanacetum trifoliolatum Podl. | NW Iran | 1868 | 1 |
| Tanacetum turcomanicum (Krasch.) Tzvel. | Lowland | 1383 | 2 |
| Tanacetum walteri (C. Winkl.) Tzvel. | Kopet Dagh | 1664 | 4 |
| Taraxacum azerbaijanicum Soest | Alborz, NW Iran | 1695 | 8 |
| Taraxacum baluchistanicum Soest | Lowland | 1073 | 1 |
| Taraxacum darbandense Soest | Alborz | 1818 | 3 |
| Taraxacum hepaticolor Soest | Zagros | 2042 | 1 |
| Taraxacum hydrophilum Soest | Lowland | 1361 | 3 |
| Taraxacum iranicum Soest | Alborz, NW Iran, Kopet Dagh | 2331 | 8 |
| Taraxacum kurdiciforme Hagl | NW Iran | 1800 | 1 |
| Taraxacum leonardii Soest | Yazd-Kerman | 2264 | 1 |
| Taraxacum neo-spurium Soest | Alborz, NW Iran | 3049 | 5 |
| Taraxacum persicum Soest | Alborz | 2558 | 2 |
| Taraxacum plicatulum Soest | Zagros | 1546 | 2 |
| Taraxacum primigenium Hand.-Mzt. | NW Iran, Yazd-Kerman | 2744 | 8 |
| Taraxacum rechingeri Soest | Alborz, Zagros | 2364 | 6 |
| Taraxacum ruberuliforme Soest | Alborz, NW Iran | 2288 | 4 |
| Taraxacum vagum Soest | Alborz | 1932 | 1 |
| Tragopogon acanthocarpus Boiss. | Alborz, Zagros, NW Iran | 2185 | 27 |
| Tragopogon bakhtiaricus Rech.f. | Zagros | 2425 | 1 |
| Tragopogon bornmuelleri M. Ownbey & Rech.f. var. latifolius Safavi & Maroofi | Zagros | 1795 | 2 |
| Tragopogon caricifolius Boiss. | Alborz, Zagros, NW Iran, Yazd-Kerman, Makran | 1949 | 70 |
| Tragopogon erostris Boiss. & Hausskn. | Zagros | 3052 | 1 |
| Tragopogon gongylorrhizus Rech.f. | Lowland | 682 | 10 |
| Tragopogon jezdianus Boiss. & Buhse | Alborz, Zagros, Yazd-Kerman, Kopet Dagh | 2184 | 10 |
| Tragopogon kotschyi Boiss. | Alborz, NW Iran | 3363 | 82 |
| Tragopogon kurdicus Safavi & Maroofi | Zagros | 2884 | 1 |
| Tragopogon maturatus Boiss. | Lowland | 1292 | 1 |
| Tragopogon porphyrocephalus Rech.f. | Alborz, Zagros, NW Iran | 1660 | 5 |
| Tragopogon rechingeri M. Ownbey | Zagros, NW Iran | 2238 | 9 |
| Tragopogon rezaiyensis Rech.f. | NW Iran, Kopet Dagh | 1886 | 8 |
| Tragopogon stroterocarpus Rech.f. | Lowland | 1355 | 2 |
| Tricholepis edmondsonii Rech.f. | Lowland | 1375 | 1 |
| Zoegea crinita Boiss. subsp. crinita | Zagros, NW Iran | 1463 | 22 |
| Zoegea leptaurea Boiss. subsp. mianensis (Boiss.) Rech.f. | Alborz, Zagros, NW Iran | 1608 | 39 |

**Table S1.2** List of taxa that have been published after the Flora Iranica (Rechinger, 1963–2015) and the Flora of Iran (Assadi, Khatamsaz, Maassoumi & Mozaffarian, 1989–2015).

| **Taxon** | **Year** | **Source** |
| --- | --- | --- |
| Centaurea ravansarensis Ranjbar & Negaresh | 2013 | Nordic Journal of Botany 30: 1–8. |
| Centaurea shahuensis Ranjbar & Negaresh | 2013 | Nordic Journal of Botany 30: 1–8. |
| Chondrilla juncea L. var. longifolia Nasseh | 2010 | Iranian Journal of Botany 16 (1): 91–95. |
| Cousinia ghahremanii Mirtadzadini & Attar | 2009 | Iranian Journal of Botany 15 (2): 146–152. |
| Cousinia argentea Mehregan & Assadi | 2009 | Willdenowia 39(2): 268. |
| Cousinia azerbaidjanica Djavadi, Attar & Najafi | 2007 | Iranian Journal of Botany 13 (1): 43–46. |
| Cousinia barezica Assadi | 2009 | Iranian Journal of Botany 15 (1): 36–44. |
| Cousinia cordifolia Djavadi & Attar | 2006 | Feddes Repertorium 117: 453–458. |
| Cousinia gatchsaranica I.Mehregan, M. Assadi & F. Attar | 2003 | Willdenowia 33: 107–111. |
| Cousinia isfahanica Assadi | 2009 | Iranian Journal of Botany 15 (1):36–44. |
| Cousinia karkasensis Mehregan & Djavadi | 2010 | Iranian Journal of Botany 16 (2): 200–203. |
| Cousinia khorasanica Djavadi & Attar | 2006 | Rostaniha 7(2): 165–175. |
| Cousinia longibracteata Attar & Mirtadzadini | 2009 | Iranian Journal of Botany 15 (2): 146–152. |
| Cousinia maassoumii Assadi | 2009 | Iranian Journal of Botany 15 (1): 36–44. |
| Cousinia mehreganii Assadi | 2011 | Iranian Journal of Botany 17 (1): 6–9. |
| Cousinia mozdouranensis Djavadi & Attar | 2005 | Feddes Repertorium 116: 285–289. |
| Cousinia papillosa Djavadi & Attar | 2007 | Rostaniha 8(2): 65–73. |
| Cousinia persica Djavadi & Attar | 2006 | Feddes Repertorium 117: 453–458. |
| Cousinia pseudocandolleana Assadi | 2009 | Iranian Journal of Botany 15 (1): 36–44. |
| Cousinia saloukensis Mehregan | 2011 | Willdenowia 41: 261–265. |
| Cousinia subpectinata Mirtadzadini, Attar & Assadi | 2004 | Iranian Journal of Botany 10 (2): 143–146. |
| Heteroderis pusilla (Boiss.) Boiss. var. khorassanica Nasseh | 2010 | Iranian Journal of Botany 16 (1): 91–95. |
| Hieracium piranshahricum Tavakkoli & Assadi | 2005 | Iranian Journal of Botany 11 (1): 59–63. |
| Jurinea cartilaginea Mozaff. | 1988 | Iranian Journal of Botany 4 (1): 61–70. |
| Klasea nana Ranjbar & Negaresh | 2012 | Annales Botanici Fennici 49: 402–406. |
| Klasea sanandajensis Ranjbar & Negaresh | 2012 | Feddes Repert 122(7-8): 466. |
| Lactuca denaensis N. Kilian & Djavadi | 2012 | PhytoKeys 11: 61–77. |
| Lactuca hazaranensis Djavadi & N. Kilian | 2012 | PhytoKeys 11: 61–77. |
| Myopordon damavandica Mozaff. | 1991 | Iranian Journal of Botany 5 (1): 29–39. |
| Psephellus khalkhalensis Ranjbar & Negaresh | 2014 | Phytotaxa 170 (3): 201. |
| Scorzonera luristanica Rech. f. var. lanata Safavi | 2006 | Iranian Journal of Botany 12 (1): 59–62. |
| Senecio eligulatus B. Nord., Moussavi & Djavadi | 2002 | Compositae Newsletter 38: 42–46. |
| Senecio subnivalis Y. Ajan, J. Noroozi & B. Nord. | 2010 | Compositae Newsletter 48: 43–62. |
| Tanacetum bachtiaricum Mozaff. | 2005 | Iranian Journal of Botany 11 (1): 115–127. |
| Tanacetum elbursense Mozaff. | 2005 | Iranian Journal of Botany 11 (1): 115–127. |
| Tragopogon bornmuelleri M. Ownbey & Rech. f. var. latifolius Safavi & Maroofi | 2006 | Iranian Journal of Botany 12 (1): 99–100. |
| Tragopogon kurdicus Safavi & Maroofi | 2014 | Iranian Journal of Botany 20 (1): 05–07. |

**REFERENCES**

Assadi, M., Khatamsaz, M., Maassoumi, A. A., & Mozaffarian, V. (1989–2015). *Flora of Iran*. Tehran: Research Institute of Forests & Rangelands.

Rechinger, K. H. (1963–2015). *Flora Iranica*. Graz & Wien: Akademische Druck- und Verlagsanstalt & Naturhistorisches Museum Wien.

**Table S1.3** Endemic taxa contributing to the score of a consensus area of endemism.

Consensus area **Zagros** (based on 17 sets), 69 species give score (endemicity score for each taxon is mentioned):

Anthemis lorestanica Iranshahr (24): (0.000-0.417)

Centaurea amadanensis Schultz-Bip. var amadanensis (44): (0.000-0.450)

Centaurea amadanensis Schultz-BIP. var gymnoclada (Jaub. & Spech) Wagenitz (45): (0.000-0.458)

Centaurea aucheri (DC.) Wagenitz subsp. farsistanica Wagenitz (48): (0.000-0.611)

Centaurea gaubae (Bornm.) Wagenitz (60): (0.000-0.464)

Centaurea koeieana Bornm. subsp. koeieana (76): (0.000-0.625)

Centaurea luristanica Rech.f. (80): (0.000-0.550)

Centaurea pterocaula Trautv. subsp. iranica Wagenitz (93): (0.000-0.556)

Centaurea xeranthemoides Rech.f. (104): (0.000-0.688)

Cousinia albescens C. Winkl. & Strauss (127): (0.000-0.700)

Cousinia albida DC. (128): (0.000-0.700)

Cousinia arakensis Attar & Djavadi (134): (0.000-0.688)

Cousinia archibaldii Rech.f. (136): (0.000-0.714)

Cousinia bachtiarica Boiss. & Hausskn. (146): (0.000-0.786)

Cousinia bazoftensis Attar (149): (0.000-0.667)

Cousinia boyerahmadica Rastegar, Attar & Mirtadz. (155): (0.000-0.600)

Cousinia calcitrapa Boiss. var. interrupta Heimerl (157): (0.000-0.450)

Cousinia calocephala Jaub. & Spech subsp. astrocephala (Hausskn. & Bornm.) Mehregan (158): (0.000-0.458)

Cousinia candolleana Jaub. & Spech (162): (0.000-0.667)

Cousinia chlorosphaera Bornm. (168): (0.000-0.833)

Cousinia concinna Boiss. & Hausskn. (171): (0.000-0.688)

Cousinia curvibracteata Mehregan (177): (0.000-0.667)

Cousinia denaensis Attar & Djavadi (184): (0.000-0.714)

Cousinia eriobasis Bunge (197): (0.000-0.346)

Cousinia hergtiana Bornm. (225): (0.000-0.700)

Cousinia iranica C. Winkl. & Strauss. (233): (0.000-0.700)

Cousinia kornhuberi Heimerl (248): (0.000-0.625)

Cousinia kotschyi Boiss. subsp. khansarica (Attar & Ghahreman) Mehregan (250): (0.000-0.667)

Cousinia lurorum (Bornm.) Bornm. (262): (0.000-0.600)

Cousinia maassoumii Assadi (263): (0.000-0.700)

Cousinia noeana Boiss. (275): (0.000-0.458)

Cousinia oligocephala Boiss. (278): (0.000-0.700)

Cousinia orthoclada Hausskn. & Bornm. (281): (0.000-0.550)

Cousinia oshtorankuhensis Attar (282): (0.000-0.700)

Cousinia pugionifera Jaub. & Spech (297): (0.000-0.455)

Cousinia sagittata C.Winkl. & Strauss subsp. iranica (C. Winkl. & Strauss) Mehregan (C. Winkl. & St (310): (0.000-0.714)

Cousinia sagittata C.Winkl. & Strauss subsp. sagittata (311): (0.000-0.917)

Cousinia silyboides Jaub. & Spach subsp. silyboides (326): (0.000-0.900)

Cousinia silyboides Jaub. & Spach subsp. zardkuhensis (Attar & Ghahreman) Mehregan (327): (0.000-0.643)

Cousinia thamnodes Borss. & Hausskn. (340): (0.000-0.800)

Crepis elymaitica Bornm. (358): (0.000-0.714)

Crepis straussii Bornm. (367): (0.000-0.500)

Echinops bakhtiaricus Rech.f. (380): (0.000-0.500)

Echinops cyanocephalus Boiss. (385): (0.000-0.700)

Echinops elymaitica Bornm. (392): (0.000-0.500)

Echinops erioceras Bornm. (394): (0.000-0.800)

Echinops hebelepis DC. (400): (0.000-0.700)

Echinops iranshahrii Rech.f. (403): (0.000-0.700)

Echinops khuzistanicus Mozaff. (410): (0.000-0.667)

Echinops kotschyi Boiss. (413): (0.000-0.800)

Echinops macrophyllus Boiss. & Hausskn. var. macrophyllus (420): (0.000-0.344)

Echinops macrophyllus Boiss. & Hausskn. var. laciniatus Mozaff. (421): (0.000-0.533)

Echinops polychromus Rech.f. (427): (0.000-0.750)

Echinops psammophilus Mozaff. (430): (0.000-0.500)

Echinops viscidulus Mozaff. (439): (0.000-0.857)

Helichrysum artemisioides Boiis. & Hausskn. (448): (0.000-0.550)

Helichrysum athanaton Georgiadou & Rech.f. (449): (0.000-0.667)

Hieracium cheirifolium Boiss. & Hausskn. (465): (0.000-0.667)

Jurinea cordata Boiss. & Hausskn. (476): (0.000-0.667)

Jurinea eriobasis DC. (477): (0.000-0.500)

Jurinea viciosoi Pau (496): (0.000-0.643)

Lactuca polyclada Boiss. (511): (0.000-0.625)

Myopordon persicum Boiss. (521): (0.000-0.700)

Pentanema multicaule Boiss. (524): (0.000-0.900)

Postia bombycina Boiss. & Hausskn. (531): (0.000-0.500)

Scorzonera ispahanica Boiss. (547): (0.000-0.708)

Scorzonera psychrophila Boiss. & Hausskn. (557): (0.000-0.700)

Scorzonera subaphylla Boiss. (562): (0.000-0.900)

Tanacetum dumosum Boiss. (584): (0.000-0.643)

Consensus area **Azerbaijan** (from 4 sets), 17 species give score (endemicity score for each taxon is mentioned):

Anthemis atropatana lranshahr (13): (0.393-0.625)

Anthemis moghanica Iranshahr (27): (0.333-0.600)

Centaurea albonitens Turrill (43): (0.000-0.433)

Centaurea congesta Wagenitz (55): (0.000-0.591)

Cousinia boissieri Buhse (155): (0.000-0.611)

Cousinia gilliatii Rech.f. (215): (0.611-0.667)

Cousinia macrocephala C. A. Mey. (264): (0.000-0.600)

Cousinia microcephala C. A. Mey. (269): (0.000-0.750)

Cousinia tabriziana Bunge (334): (0.722-0.833)

Cousinia tenuifolia C. A. Mey. (336): (0.500-0.667)

Cousinia urumiensis Bornm. (345): (0.458-0.591)

Hieracium azerbaijanense Lack (464): (0.000-0.667)

Jurinea leptoloba DC. (485): (0.667-0.750)

Jurinea multicaulis DC. (490): (0.500)

Jurinella moschus (Habl.) Bobrov. (499): (0.000-0.368)

Scorzonera szovitsii DC. (563): (0.625-0.857)

Senecio lipskyi Lomak. (570): (0.667-0.750)

Consensus area **Kopet Dagh** (from 2 sets), 19 species give score (endemicity score for each taxon is mentioned):

Centaurea galactochora Rech.f. (59): (0.000-0.667)

Cousinia adenostegia Rech.f. (123): (0.667)

Cousinia argentea Mehregan & Assadi (139): (0.000-0.667)

Cousinia chaetocephala Kult. (165): (0.563-0.833)

Cousinia dipterocarpa Bornm. & Rech.f. (187): (0.667)

Cousinia eriophylla (Kult.) Bornm. (198): (0.667)

Cousinia komarowii (O. Kuntze) C. Winkl. (246): (0.500-0.750)

Cousinia lasiandra Bunge (253): (0.333-0.450)

Cousinia platyacantha Bunge (293): (0.667)

Cousinia trachyphyllaria Bornm. & Rech. f . (343): (0.000-0.667)

Cousinia verbascifolia Bunge (346): (0.300)

Echinops heteromorphus Bunge (401): (0.500-0.750)

Echinops procerus Mozaff. (429): (0.000-0.667)

Jurinea catharinae Iljin. (475): (0.000-0.400)

Launaea peistocarpa (Boiss.) Rech.f. (514): (0.750)

Tanacetum khorassanicum (Krasch.) Parsa (588): (0.667)

Tanacetum turcomanicum (Krasch.) Tzvel. (600): (0.667)

Tanacetum walteri (C. Winkl.) Tzvel. (601): (0.000-0.667)

Taraxacum hydrophilum Soest (606): (0.000-0.500)

Consensus area **Alborz** (from 3 sets), 26 species give score (endemicity score for each taxon is mentioned):

Anthemis altissima L. var. discoidea Iranshahr (12): (0.000-0.750)

Anthemis triumfettii (L). All. subsp. khorasanica (Rech.f.) lranshahr (33): (0.000-0.571)

Carduus transcaspicus Gandog. subsp. macrocephalus (Arenes) Kazmi (42): (0.000-0.563)

Centaurea lachnopus Rech.f. (78): (0.000-0.750)

Cephalorrhynchus gorganicus (Rech.f. & Esfand.) Tuisl (108): (0.500-0.688)

Cousinia calocephala Jaub. & Spech subsp. behboudiana (Rech.f. & Esfand.) Mehregan (159): (0.000-0.750)

Cousinia commutata Bunge (170): (0.000-0.500)

Cousinia firuzkuhensis Rech.f. (205): (0.000-0.750)

Cousinia glaucopsis Bornm. & Rech.f. (216): (0.000-0.875)

Cousinia meluarmanica Rech.f. (267): (0.000-0.750)

Cousinia nekarmanica Rech.f. (273): (0.000-0.688)

Cousinia shahvarica Rech.f. (321): (0.000-0.650)

Cousinia xiphiolepis Boiss. (349): (0.000-0.750)

Crepis asadbarensis Bornm. ex Rech.f. (353): (0.000-0.500)

Crepis ciliata C. Koch (354): (0.000-0.750)

Crepis demavendi Bornm. (356): (0.000-0.750)

Crepis willemetioides Boiss. (368): (0.000-0.571)

Doronicum wendelboi Edmondson (374): (0.000-0.688)

Echinops nizvanus Rech.f. (423): (0.000-0.688)

Erigeron hyrcanicus Bornm. & Vierh. (443): (0.300-0.750)

Erigeron uniflorus L. subsp. elbursensis (Boiss.) Rech.f. (445): (0.500-0.688)

Iranecio elbrusensis (Boiss.) B. Nord. (470): (0.300-0.750)

Jurinella frigida (Boiss.) Wagenitz (497): (0.000-0.375)

Ligularia persica Boiss. (517): (0.000-0.375)

Tanacetum tenuisectum (Boiss.) Podl. (598): (0.000-0.667)

Tragopogon gongylorrhizus Rech.f. (622): (0.000-0.750)

Consensus area **Central Alborz** (from 1 set), 24 species give score (endemicity score for each taxon is mentioned):

Achillea millefolium L. subsp. elbursensis Hub.-Mor. (5): (0.583)

Centaurea kandavanensis Wagenitz (73): (0.500)

Cousinia adenosticta Bornm. (124): (0.750)

Cousinia akredii Bornm. & Gauba (126): (0.750)

Cousinia calocephala Jaub. & Spech subsp. behboudiana (Rech.f. & Esfand.) Mehregan (159): (0.750)

Cousinia chamaepeuce Boiss. (166): (0.875)

Cousinia commutata Bunge (170): (0.500)

Cousinia crispa Jaub. & Spech (176): (0.875)

Cousinia gaubae Bornm. (211): (0.750)

Cousinia gmelini C. Winkl. (217): (0.500)

Cousinia sphaerocephala Jaub. & Spech (329): (0.750)

Cousinia xiphiolepis Boiss. (349): (0.750)

Crepis asadbarensis Bornm. ex Rech.f. (353): (0.875)

Crepis ciliata C. Koch (354): (0.750)

Crepis demavendi Bornm. (356): (0.750)

Crepis heterotricha DC. subsp. lobata Babcock (361): (0.750)

Echinops elbursensis Rech.f. (391): (0.750)

Jurinea macrocephala DC. subsp. elbuesensis Wage (486): (0.750)

Jurinella frigida (Boiss.) Wagenitz (497): (1.000)

Ligularia persica Boiss. (517): (1.000)

Scorzonera kandavanica Rech.f. (549): (0.750)

Senecio vulcanicus Boiss. (572): (0.750)

Tanacetum hololeucum (Bornm.) Podl. (586): (0.750)

Taraxacum darbandense Soest (604): (0.750)


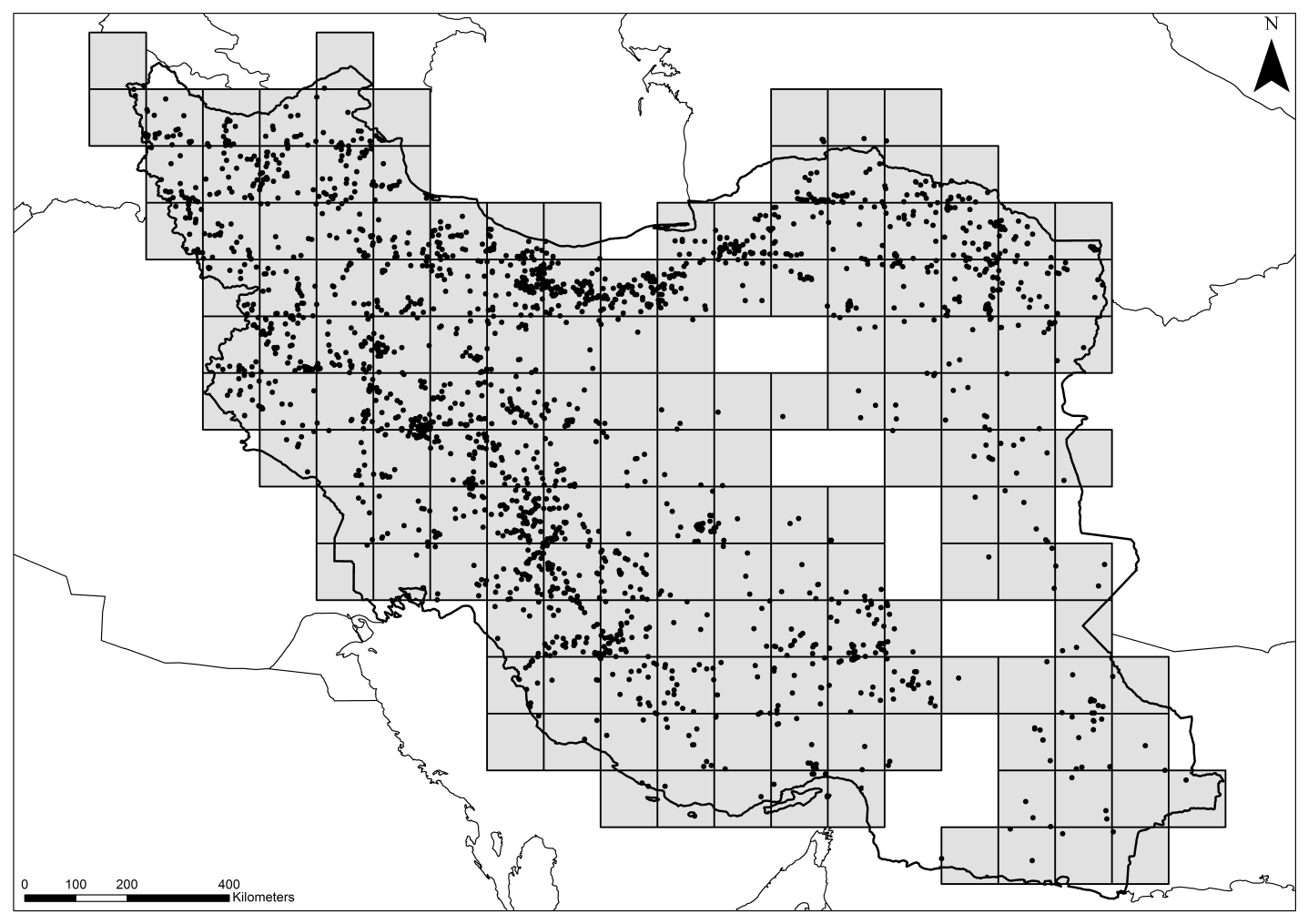


**Figure S1.1** Geographic distribution of the 5,984 used Asteraceae records.


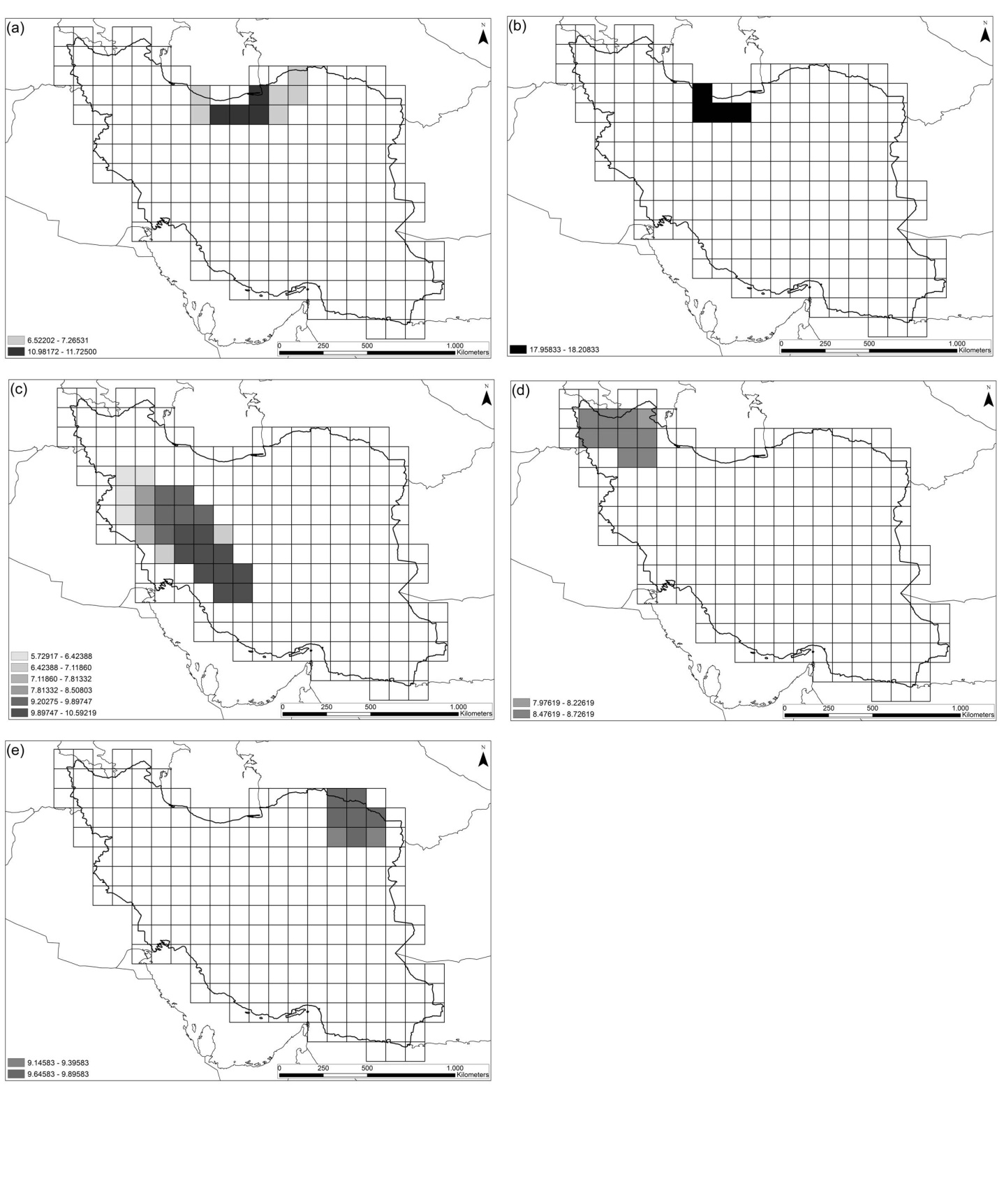


**Figure S1.2** Areas of endemism in Iran: (a) Alborz. (b) Central Alborz. (c) Zagros. (d) Azerbaijan. (e) Kopet Dagh-Khorassan. Classes of endemicity scores are indicated by different shades of grey.


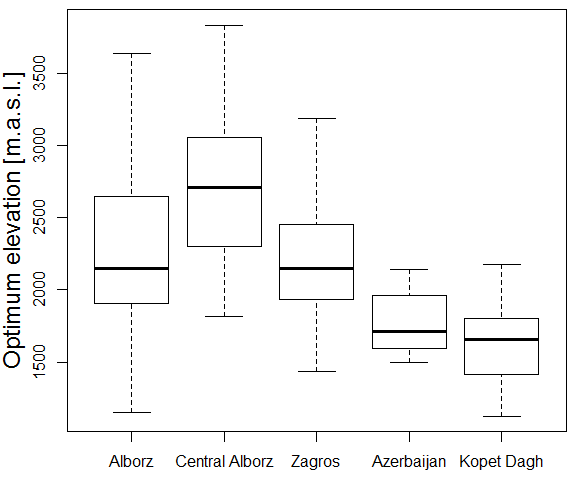


**Figure S1.3** Optimum elevations of taxa supporting an area of endemism. Boxes represent the interquartile range and horizontal lines the median.
